# Integrated computational strategies for Polypharmacological profiling and identification of anti‐inflammatory targets in *Rungia pectinata* L.

**DOI:** 10.1111/jcmm.70158

**Published:** 2024-12-04

**Authors:** Alaiha Zaheen, Sanchaita Rajkhowa, Sami A. Al‐Hussain, Magdi E. A. Zaki

**Affiliations:** ^1^ Centre for Biotechnology and Bioinformatics Dibrugarh University Dibrugarh India; ^2^ Department of Chemistry Imam Mohammad Ibn Saud Islamic University (IMSIU) Riyadh Saudi Arabia

**Keywords:** anti‐cancer, anti‐inflammatory, molecular dynamics simulation, network pharmacology, *Rungia pectinata* L.

## Abstract

*Rungia pectinata* L. is an ethnomedicinal herb belonging to the Acanthaceae family and it presents a promising avenue for medicinal exploration, deeply rooted in traditional practices. Earlier research has demonstrated that the herb can effectively relieve the classic symptoms of inflammation. Nevertheless, comprehensive studies into the mechanisms underlying *R. pectinata*'s beneficial impact on inflammation pathways, remain scarce. Hence, we employed an integrated approach combining network pharmacology, molecular docking and molecular dynamics simulations to explore the mechanisms underlying *R*. *pectinata*'s anti‐inflammatory activity. For this study, seven inflammation‐related active ingredients were identified among 38 candidates, revealing 22 intersecting genes associated with inflammation. Protein–protein interaction (PPI) networks revealed three therapeutic targets: *IL1B*, *PTGS2* and *SRC*. GO and KEGG pathway enrichment analyses indicated that the effects of *R. pectinata* are mediated by genes related to inflammation and cancer. Molecular docking studies identified trans‐nerolidyl formate and widdrol as lead compounds while molecular dynamics simulations indicated stable compound‐target complexes, with MM‐PBSA calculations showing superior free energy values for *SRC*, suggesting implications in cancer pathways. Overall, this study offers valuable insights into the anti‐inflammatory effects of *R. pectinata*, which may be mediated through key pathways involved in inflammation and cancer. This highlights the potential of *R. pectinata* in both anti‐inflammatory and anticancer therapies. However, further experimental validation is necessary to confirm these findings.

## 1 INTRODUCTION

Medicinal plants are a vital source of pharmacologically active compounds, many of which have led to the development of novel therapeutics.^1^ Their extensive historical use in treating human diseases highlights their therapeutic potential. In recent years, the popularity of medicinal plants has increased due to their perceived safety and lower incidence of adverse effects compared to synthetic drugs.^2^, ^3^ Notably, natural products and their derivatives constitute a significant portion of current pharmaceuticals and traditional medicine remains the primary healthcare source for over 85% of the global population.^4^ The use of natural products has significantly contributed to the identification, synthesis, and development of novel lead molecules for chemotherapy, immunomodulation, drug development and biosensor design.^5^ Advances in various scientific fields have further facilitated these processes.^6^

*Rungia pectinata* L., an ethnomedicinal herb from the Acanthaceae family, also known as *Justicia pectinata, R. parviflora var. pectinata* and *R. parviflora var. muralis*, is a small, soft annual herb with lance‐shaped membranous leaves and fine blue flowers. In India, it is found in Assam, Andhra Pradesh, Karnataka, Kerala, Maharashtra, Odisha and Tamil Nadu, and it also grows in neighbouring countries such as Myanmar, China, Sri Lanka, Java, Malaysia and the Philippines. Phytosterols, terpenes, tannins, glycosides, flavonoids, phenolic compounds, amino acids and fixed oils are key phytochemicals isolated from this plant.^7^ According to ‘Indian Materia Medica’ by Nadkarni et al.,^8^ leaf juice from the plant is used as a cooling agent and to cure smallpox in infants. Bruised leaves are applied externally to relieve painful inflammations and swellings and fresh leaf paste mixed with castor oil is used to treat tinea capitis. The roots are employed as febrifuge and vermifuge by tribal populations in India. Additionally, *R. pectinata* exhibits antipyretic, anti‐inflammatory, diuretic, analgesic, antifungal and antimicrobial activities, as supported by traditional use and experimental studies.^8^

Despite *R. pectinata's* documented efficacy in alleviating inflammation symptoms,^9^ comprehensive studies investigating its impact on inflammation pathways, specific biological targets and metabolic processes are limited. Thus, this research aims to address this gap by identifying active ingredients and their corresponding targets, constructing protein–protein interaction networks and conducting enrichment analyses to map out the involved biological pathways.

Chronic inflammation plays a crucial role in tumour development by promoting cell proliferation, survival and migration, which significantly elevates neoplasm risk. Understanding the molecular mechanisms of anti‐inflammatory agents like *R. pectinata* is therefore essential. Network pharmacology, combined with molecular docking and molecular dynamics (MD) simulations, was employed in this study to predict the bioactive compounds, potential targets and signalling pathways involved in *R. pectinata*'s anti‐inflammatory effects. This integrated computational strategy provides a comprehensive understanding of its mechanisms.

## 2 MATERIALS AND METHODS

**FIGURE 1:**
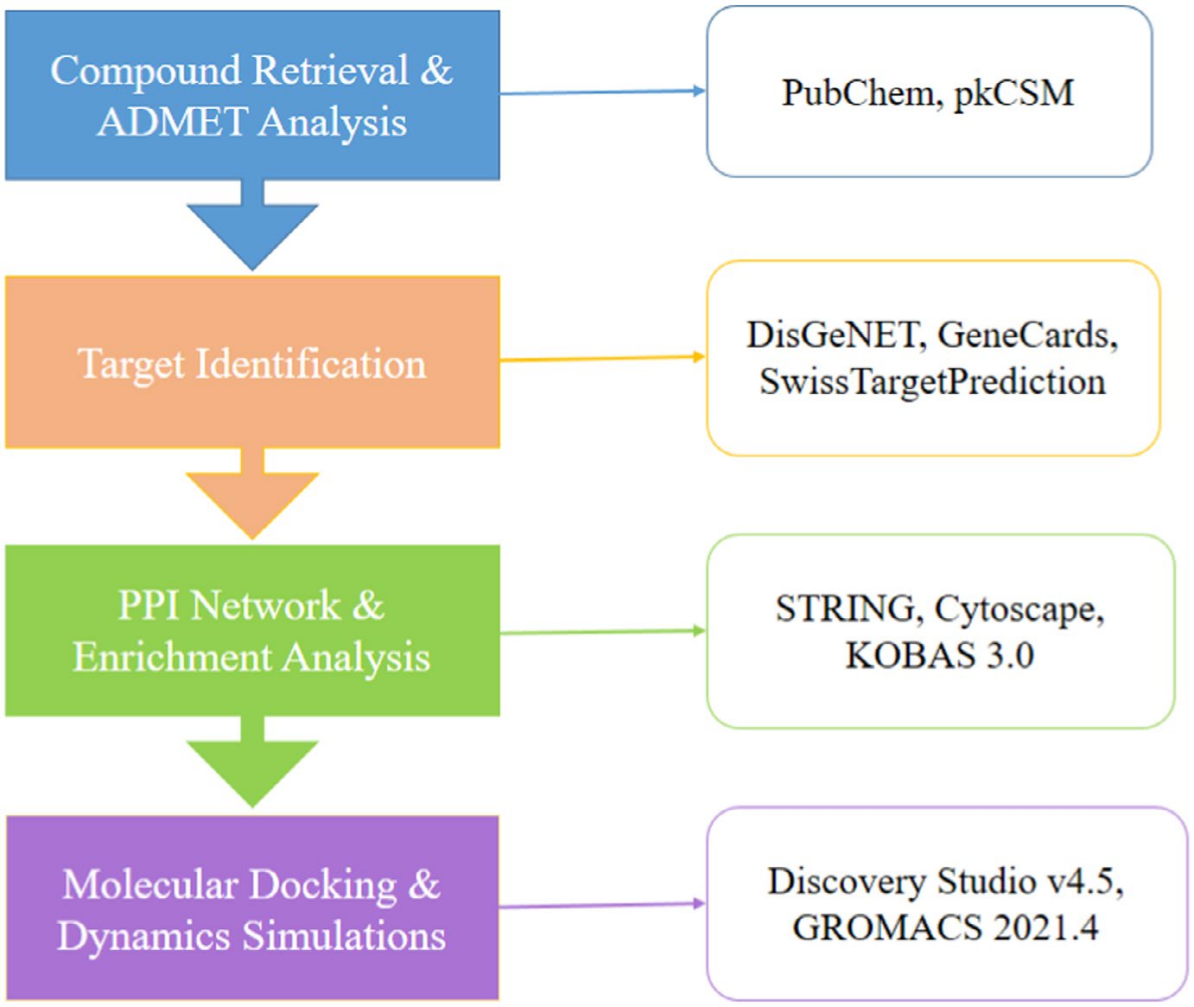
The workflow of the present study.

The current study's methodology is integrative and involves a comprehensive series of steps, as shown in Figure 1. Initially, active constituents of *Rungia pectinata* L. were collected through a literature mining approach, focusing on chemical structures and biological activities. The identified compounds were then subjected to ADMET and drug‐likeness analysis using the pkCSM server, ensuring the selection of candidates with favourable pharmacokinetic profiles. SwissTargetPrediction was utilized to analyse corresponding targets in *R. pectinata*. Subsequently, potential anti‐inflammatory targets were acquired by cross‐referencing inflammation‐related proteins from DisGeNET and GeneCards databases with proteins regulated by the bioactive compounds. A PPI network was constructed using the STRING database to visualize the interactions between these targets, further refined in Cytoscape. GO and KEGG enrichment analyses were performed to gain insights into the molecular functions, biological processes and pathways associated with the target gene products. Following this, binding site prediction and molecular docking were carried out using Discovery Studio, focusing on the core target proteins. Finally, MD simulations were performed with GROMACS to assess the stability and flexibility of the protein‐ligand interactions over time.

### 2.1 Collection of active constituents and ADMET analysis

A literature mining approach identified key studies, particularly Zhang et al., on the chemical constituents of *R. pectinata*.^10^ Essential data, including 3D structures, molecular properties and PubChem identifiers, were retrieved from the PubChem database, excluding compounds without complete identifiers.^11^

ADMET analysis is widely acknowledged as the primary screening method for assessing the suitability of bioactive molecules in virtual screening. Accurately predicting ADMET factors (Absorption, Distribution, Metabolism, Excretion and Toxicity) during early drug development is crucial for evaluating the potential of bioactive compounds. The drug‐likeness of compounds within the library was assessed by evaluating their central ADMET properties using the open‐source online webserver pkCSM.^12^ In our screening process, we established specific criteria to evaluate the potential of each compound. The standards we used include adherence to Lipinski's rule of five, which helps determine the drug‐likeness of the compounds.^13^ This rule assesses properties such as molecular weight, lipophilicity (logP), hydrogen bond donors and acceptors and the number of rotatable bonds, all of which are indicative of the compound's oral bioavailability and general pharmacokinetic behaviour. Additionally, we assessed key pharmacokinetic parameters relevant to physiological values, including intestinal absorption and permeability across biological membranes. High intestinal absorption (HIA) values were used as a standard to gauge the compound's potential for effective oral delivery. We also evaluated the compounds' permeability across the blood–brain barrier (BBB) and their overall distribution characteristics, including the volume of distribution at steady state (VDss), which reflects how extensively the drug disperses into body tissues. pkCSM's use of graph‐based signature methods allows for a detailed analysis of compound properties, aligning with established rule sets such as Lipinski and Veber criteria to determine drug‐likeness. Moreover, the integration of predictive models within pkCSM, such as those for mutagenicity and rodent carcinogenicity, enhances the assessment of compound safety and efficacy profiles.^14^

These standards were chosen based on their relevance to predicting the compounds' efficacy and safety profiles in physiological conditions. By focusing on these criteria, we aimed to identify compounds with favourable pharmacokinetic properties and reduced likelihood of adverse effects, which are crucial for their potential therapeutic applications. Additionally, the ADMET predictions provided insights into potential metabolic pathways and excretion routes, which are critical for understanding how long a compound will remain active in the body and how frequently it needs to be administered.

### 2.2 Acquisition of potential anti‐inflammatory targets

Following this, natural compounds meeting ADMET criteria and demonstrating drug‐like properties were selected for further evaluation of potential therapeutic targets.

The DisGeNET database contains comprehensive information on genes and mutation sites associated with human diseases.^15^, ^16^ Similarly, GeneCards provides detailed genomic, proteomic, transcriptional, genetic and functional data for all known and predicted human genes.^17^, ^18^ These two databases were utilized to create inflammation‐related target libraries through automated searches using keywords like “inflammation” and “inflammatory.” The search results underwent additional filtering based on DisGeNET score (gda ≥0.2) and GeneCards score (≥20).^19^ Targets related to inflammation were then cross‐referenced with proteins regulated by bioactive compounds of *R. pectinata*, using bioactive‐regulated proteins from SwissTargetPrediction. The shared targets between the composition of *R. pectinata* and inflammation‐related proteins were identified using the Venn Diagram Tool v2.1.0.^20^ This approach integrates information from disease‐associated genes (DisGeNET), known human genes (GeneCards) and predicted bioactive targets of *R. pectinata* to identify potential targets relevant to inflammation. The use of specific filtering criteria ensures the selection of high‐confidence targets associated with inflammatory processes. The Venn diagram analysis facilitates the visualization and identification of overlapping targets, highlighting potential intersections between the bioactive components of *R. pectinata* and inflammation‐related pathways or proteins. This systematic methodology aids in prioritizing targets for further investigation in the context of inflammation‐related therapeutic interventions.

### 2.3 Construction of a PPI Network

The construction of a PPI network for potential targets was conducted using the STRING database.^21^ This database allows the creation of a PPI network by entering either a single protein name, multiple names, or an amino acid sequence in the STRING website, facilitating the visualization of protein interactions. Targets from a dataset intersecting with inflammation targets were mapped, and a PPI network was constructed based on STRING v12.0 database information.^22^ This network focused on ‘*Homo sapiens*’ and was filtered with a medium confidence score of 0.7. To ensure the completeness and accuracy of the PPI network analysis, TSV format files generated from STRING were imported into Cytoscape 3.7.1 software.^23^

To elucidate the anti‐inflammatory mechanisms of *R. pectinata* from a network target perspective, we created two types of visualized networks: a target‐protein–protein interaction network and an herb‐compound‐target‐pathway network.^24^ In these networks, nodes represent drugs, compounds, or genes/proteins and edges represent associations between these nodes. Each node's importance was assessed based on three significant parameters: ‘Degree,’ which indicates the number of edges connected to a node; ‘Betweenness,’ which quantifies the number of shortest paths passing through a node between pairs of nodes; and ‘Closeness,’ which reflects the inverse of the sum of distances from a node to all other nodes in the network.^25^ The CytoHubba plugin within Cytoscape was employed to identify hub genes within the network, aiding in the identification of key targets for further analysis and exploration.

### 2.4 GO and KEGG enrichment analyses

Gene Ontology (GO) provides a structured framework in Bioinformatics to annotate and understand gene functions across three categories: Molecular Function (MF), Biological Process (BP) and Cellular Component (CC).^26^ The Kyoto Encyclopedia of Genes and Genomes (KEGG) complements this by mapping gene functions within molecular networks, particularly through its PATHWAY database, which visualizes key metabolic and regulatory pathways.^27^

Intersecting genes were evaluated by GO and KEGG enrichment analyses using the cytoscape plugin BINGO to gain insight into the molecular function, biological process and cellular components of the target gene products & KOBAS 3.0 which assigns potential pathways and connections to diseases to a given set of genes by associating them with genes that already have established annotations.^28^, ^29^ The main BP, CC and MF as well as significantly altered KEGG pathways were visualized using bar plots and dot plots generated with SRplot.^30^ Central targets were identified by screening for interactions between these targets and pathways.

### 2.5 Density functional theory (DFT) analysis

A DFT study was conducted on the ADMET qualified compounds using gradient‐corrected density functional theory (DFT) with DMol3 integrated within BIOVIA Discovery Studio v4.5 (DS v4.5) as outlined by SYSTEMES (Biovia, 2017). We employed a DNP basis set in conjunction with the generalized gradient approximation (GGA) exchange‐correlation functional B3LYP, which offers similar size to the 6‐31G** basis set but provides superior accuracy compared to a Gaussian basis set of equivalent size. This DFT analysis aimed to evaluate the chemical reactivity descriptors, providing insights into the chemical and biological activities of the active compounds.^31^ Determining a chemical species' reactivity requires knowledge of its HOMO‐LUMO border orbitals.^32^ A molecule's E_LUMO_ value indicates how many electrons it can gain, whereas a greater E_HOMO_ value indicates how many electrons it can donate to molecules whose unfilled orbitals have lower energies. Stability decreases and reactivity increases when the HOMO‐LUMO energy gap closes. Stability and reactivity are represented by the chemical hardness unit (η). Greater stability and reduced reactivity are characteristics of harder molecules, which have larger HOMO‐LUMO energy gaps. Conversely, a more reactive and softer molecule has a smaller energy gap.^33^ Higher electronic chemical potential molecules are typically more reactive and less stable. For all identified compounds, the energies of the frontier orbitals, specifically the Highest Occupied Molecular Orbital (HOMO) and the Lowest Unoccupied Molecular Orbital (LUMO), were calculated. Compounds with the smallest HOMO‐LUMO energy gaps were identified as potential candidates for exhibiting multiple targeted behaviours.^34^

### 2.6 Prediction of binding site and molecular docking

In proteins, binding and active sites typically correspond with structural pockets and cavities within the protein's three‐dimensional structure. The PDB structures of the proteins already included native ligands, which allowed us to use these native ligand‐binding sites as the reference binding sites for all three proteins. The binding sites for docking studies were confirmed by ensuring that the identified sites in the simulated structures corresponded with those of the native ligands in the original PDB structures. This validation was essential to maintain the structural integrity and relevance of the docking studies, ensuring that the selected binding sites were functionally and structurally appropriate which improved our understanding of the protein's spatial properties and potential binding regions. To ensure the accuracy of the binding sites, the simulated structures of the proteins were superimposed with their respective original PDB structures using PyMOL.

Molecular docking is a crucial technique for predicting the binding affinities of small molecules to proteins, generating and ranking multiple ligand‐protein complex conformations based on their affinity. The primary objective is to assess binding energy and interaction types between ligands and the target protein, aiming to identify potential drug candidates and understand ligand‐receptor interactions.

In this study, the Discovery Studios platform (DS v4.5) was employed for calculations, using the receptor‐ligand tool to prepare the protein and ligands. The three‐dimensional structures of core target proteins (*SRC*, *IL1B* and *PTGS2*) for molecular docking within the PPI network were retrieved from the Protein Data Bank. Due to missing residues, the proteins *IL1B* and *PTGS2* were modelled using SWISS‐MODEL. Compounds with optimal ADMET results were docked to the target proteins. The CDOCKER tool was used to generate and evaluate ligand conformations based on binding energy and interaction isomers. The Discovery Studio Visualizer enabled comprehensive visualization and examination of the docked structures, facilitating detailed exploration of molecular interactions and conformational aspects within the binding site.^35^

### 2.7 MD simulations

Following the identification of compounds with strong binding affinity, MD simulations were conducted to evaluate their binding stability and flexibility. These simulations assessed the stability and conformational changes of the proposed model and investigated protein‐ligand interactions over time. The simulations, lasting 50 ns for both the protein‐ligand complex and the native proteins, were performed using the GROMACS 2021.4 package.^36^ Ligand topology was generated using the Automated Topology Builder (ATB) and Repository server,^37^ supporting the Gromos 54a7 force field. The structures were solvated in a cubic water box with the SPC/E water model, and counter ions were added to neutralize net charges. The solute molecules were kept 1.0 nm from the box boundary. Energy minimization was conducted using the steepest descent method, followed by equilibration in two phases. Initially, a 2 ns NVT run stabilized the temperature, followed by a 5 ns NPT simulation to balance the pressure, maintaining constant atom number, pressure and temperature. The dynamics simulation ran in an NPT canonical ensemble at 300 K and 1 bar.^31^ Analysis of the protein's RMSD, RMSF and Rg over time was conducted using Xmgrace and MM/PBSA (Molecular Mechanics/Poisson‐Boltzmann Surface Area) analysis, integrated within GROMACS 2021.4, was used to estimate binding free energies by combining molecular mechanics energies with solvation free energies from the Poisson‐Boltzmann equation and solvent‐accessible surface area (SASA).^38^

## 3 RESULTS AND DISCUSSION

### 3.1 Screening of bioactive compounds of *R. pectinata*

Literature and database mining was carried out to compile a collection of 38 natural compounds derived from *R. pectinata*.^10^ These compounds were subsequently analysed using the pkCSM online server. The findings indicated that the seven compounds, CID608370, CID12565, CID31404, CID138824, CID617875, CID5363406 and CID94334, adhered to Lipinski's rule and could potentially meet the criteria for orally active drugs.^39^ The compounds underwent in silico ADME assessments, offering insights into their pharmacokinetic characteristics, encompassing absorption, distribution, metabolism and excretion (Table 1). In evaluating the compounds' distribution properties, diverse metrics were analysed, including volume of distribution at steady state (VDss), the fraction of unbound drug and permeability across the blood–brain barrier (BBB).

**TABLE 1:** Physicochemical properties, pharmacokinetics, druglikeness and toxicity prediction of the seven compounds.

Physicochemical properties	CID608370	CID12565	CID31404	CID138824	CID617875	CID5363406	CID94334
Molecular weight	216.324	220.268	220.356	228.420	256.389	250.382	222.372
No. of rotatable bonds (< 10)	0	2	0	9	0	9	0
No. of H‐bond Acceptor (<10)	1	3	1	1	1	2	1
No. of H‐bond Donor (<5)	0	0	1	1	0	0	1
logP(<5)	3.848	2.888	4.295	4.637	4.391	4.577	4.064
Pharmacokinetics							
Water solubility	−4.857	−2.611	−4.834	−5.923	−5.369	−5.282	−4.366
CaCo‐2 permeability	1.128	1.910	1.741	1.504	1.475	1.631	1.498
Human Intestinal Absorption (HIA)	96.074	95.280	91.904	91.753	95.643	94.357	91.951
Skin Permeability	−2.058	−2.141	−2.474	−1.829	−1.896	−1.953	−1.946
VDss Human	0.638	0.139	0.932	0.424	0.957	0.273	0.462
Blood–brain barrier (BBB)	0.427	0.097	0.434	0.747	0.436	0.601	0.553
Total Clearance	0.937	0.243	0.775	1.526	0.871	1.809	0.925
Toxicity							
Ames Toxicity	Non‐mutagen	Non‐mutagen	Non‐mutagen	Non‐mutagen	Non‐mutagen	Non‐mutagen	Non‐mutagen
hERG inhibition	No	No	No	No	No	No	No

**FIGURE 2:**
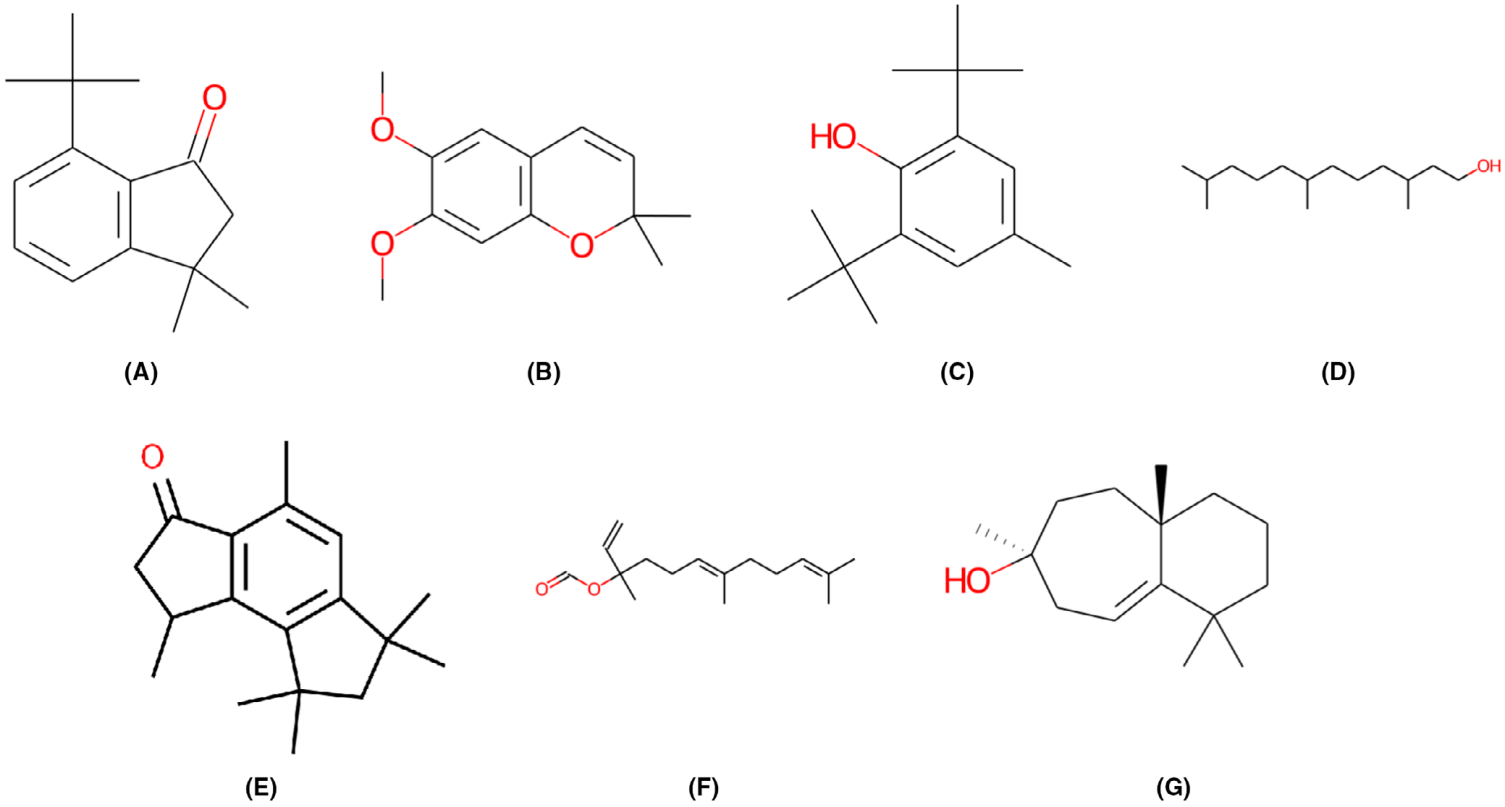
Structures of the seven ADMET qualified compounds, (A) CID608370 (B) CID12565 (C) CID31404 (D) CID138824 (E) CID617875 (F) CID5363406 (G) CID94334.

Consequently, the seven compounds exhibited favourable intestinal absorption (HIA). A higher HIA value suggests increased absorption potential when orally administered through the intestinal tract. In‐vitro studies with CaCo‐2 cells demonstrated good permeability results. Maximum skin permeability ranged from −2.058 to −1.946 for all compounds. Based on these findings, it was concluded that the compounds exhibited promising ADME properties. The total clearance of a compound represents the rate at which it is eliminated from the body. The findings from Table 1 indicated that among the seven compounds examined, CID12565 exhibited the lowest total clearance (0.243). Typically, lower total clearance is advantageous for drug candidates as it can prolong the duration of action and potentially reduce the frequency of dosing. Mutagenicity was evaluated using the AMES test, while assessment of cardiac toxicity for the compounds involved measuring hERG inhibition. CID608370, CID12565, CID31404, CID138824, CID617875, CID5363406 and CID94334 showed non‐mutagenic behaviour in the AMES test and no hERG inhibition. Among the selected compounds/ligands, these seven compounds exhibited notable ADMET properties and met the criteria for drug‐likeness scores (Table 1 and Figure 2).

**FIGURE 3:**
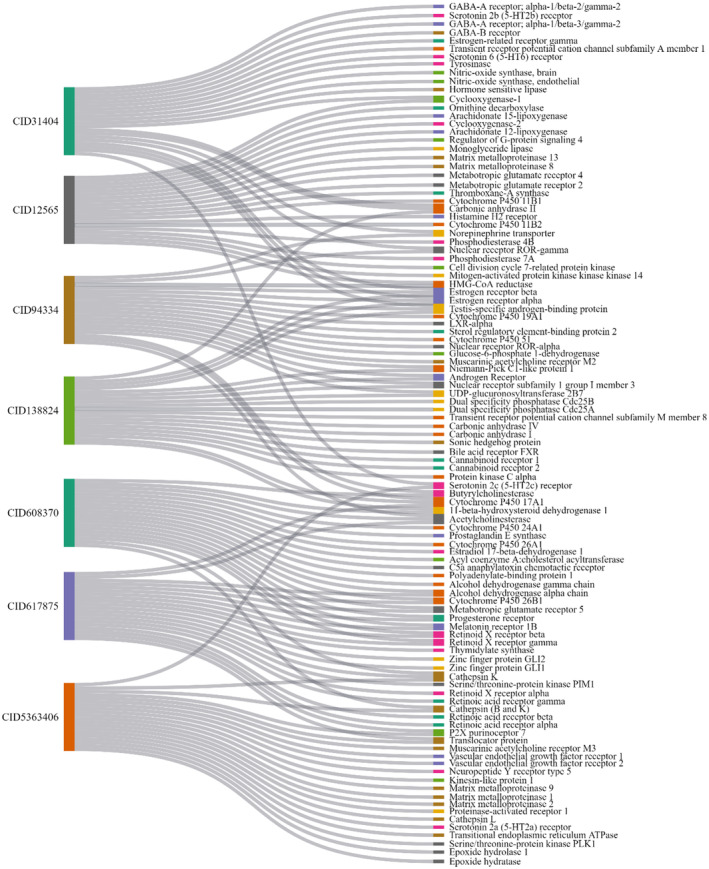
The Sankey plot showing Compound‐Target network. Left column represents the compounds and the right column represents the top 20 target proteins of each compound.

A reverse pharmacophore approach was employed to obtain the potential targets using the screened compounds as input.^40^ Top 20 hits for each of the seven compounds were selected based on the highest probability. Overall, a total of 140 ingredient‐related targets were identified using the SwissTargetPrediction database (Table ST1—Data S1). The compounds and their corresponding biological protein targets were analysed using a Sankey graph, also known as bipartite network graph, approach to identify proteins that are significantly associated with multiple compounds (Figure 3).

In this biomedical network graph, compounds and protein targets are represented as nodes belonging to two distinct sets. The set on the left represents compounds, while the set on the right represents target proteins. Connections between compounds and target proteins are depicted by grey edges.^41^

### 3.2 Acquisition of potential anti‐inflammatory genes

**FIGURE 4:**
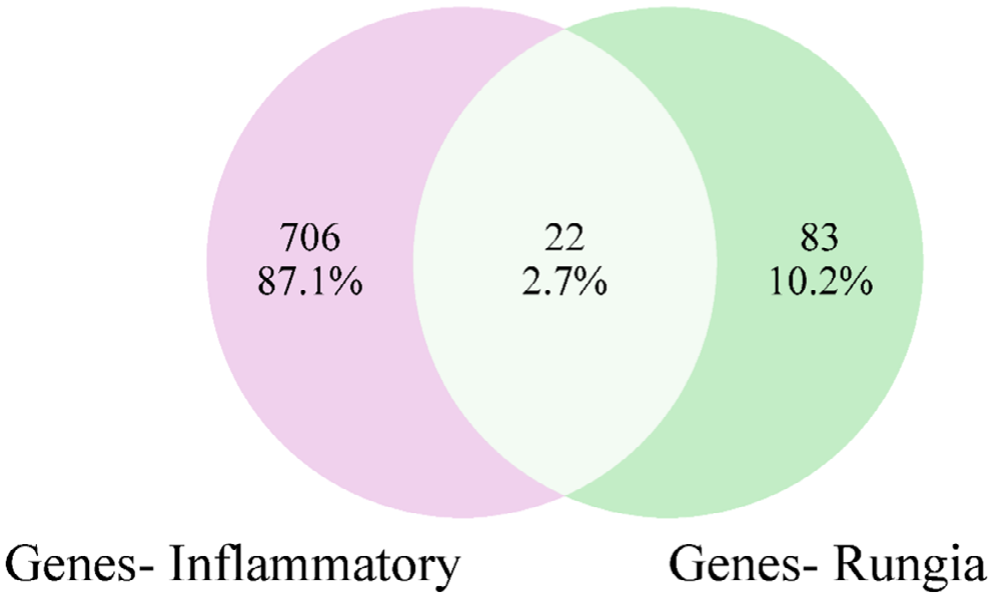
The intersection of active ingredient targets and inflammation targets resulting in 22 common targets. This Venn diagram was built using the Venn Diagram Tool v2.1.0 platform.

A total of 728 targets related to inflammation were identified from DisGeNET and GeneCards databases. The genes linked with the compounds were compared to anti‐inflammatory genes within the Venny 2.1.0 database,^20^ resulting in a gene interaction chart (Figure 4). Twenty‐two target genes of *R. pectinata* against inflammation were derived, totaling 2.7% of the genes.

### 3.3 Protein–protein interaction (PPI) network analysis

**FIGURE 5:**
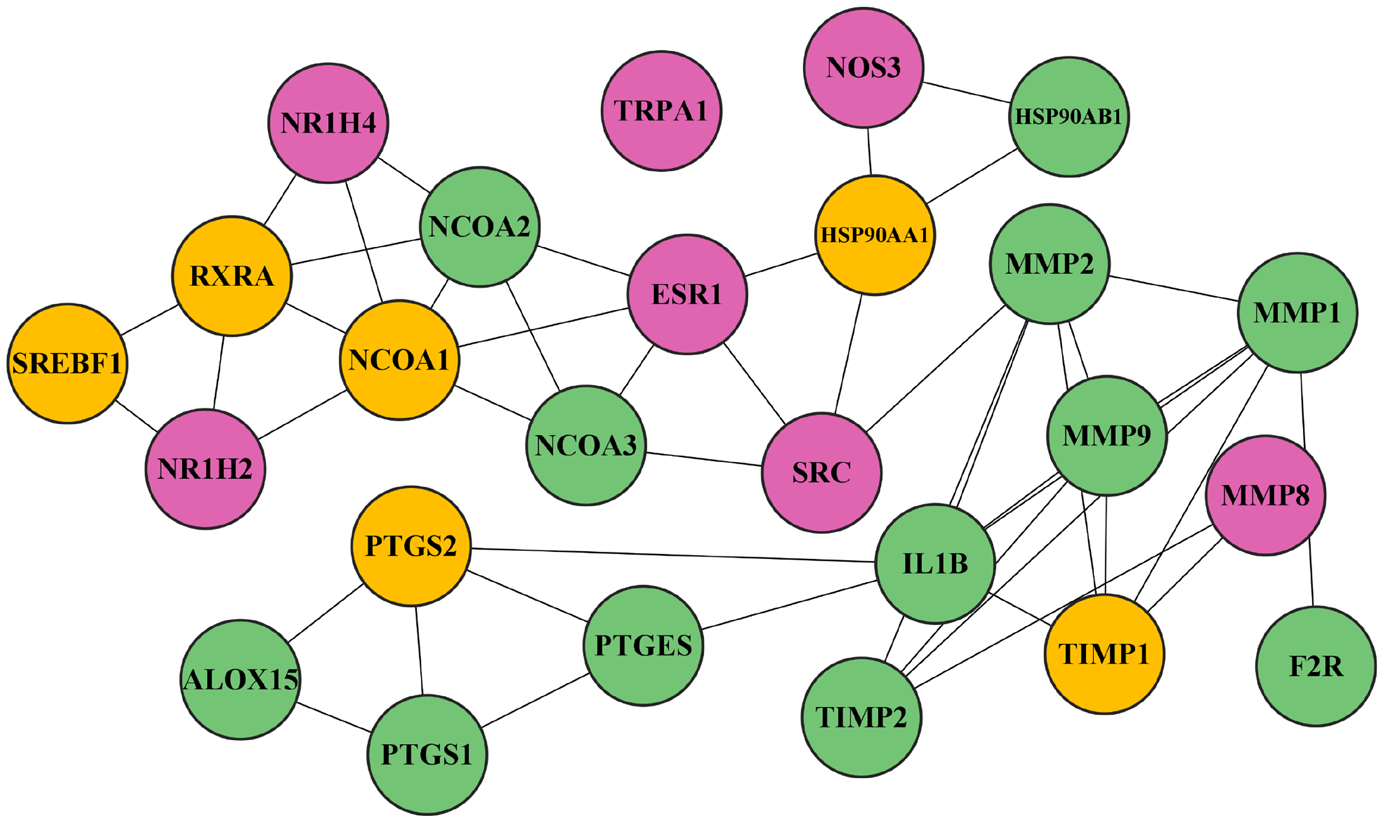
PPI Network of the top 25 hub genes. The yellow colour indicates the nodes with high degree scores, the green colour indicates the nodes with intermediate degree scores and the magenta indicates the node with low degree scores.

The specific intersection targets were identified and subjected to PPI analysis using the STRING database. Interaction networks with confidence scores greater than 0.7 were imported into Cytoscape 3.7.1 for further analysis. A total of 129 nodes and 442 edges were observed in the network. The whole network showing the core targets and non‐core targets is illustrated in Figure SF1—Data S1. The nodes represent proteins and the edges indicate their relations. The connections (edges) in the resulting network represent the degree of association between the intersectional genes. The networks were processed using the Network Analyser plugin to compute topological properties such as node degree distribution, betweenness centrality and closeness centrality. The top 25 key nodes were identified based on node degree (>9), as demonstrated in Figure 5.

Notably, the top 4 nodes in the protein interaction network (PIN) were *TIMP1* (degree = 19), *NCOA1* (degree = 18), *SREBF1* (degree = 17) and *PTGS2* (degree = 17). These superhub genes play crucial roles in forming functional networks that contribute to cellular activities. Excluding these proteins disrupts interactions, potentially impacting normal cellular functions.^42^ The detailed list of top genes involved in the PIN is provided in the table ST2—Data S1.

### 3.4 Functional enrichment analysis

**FIGURE 6:**
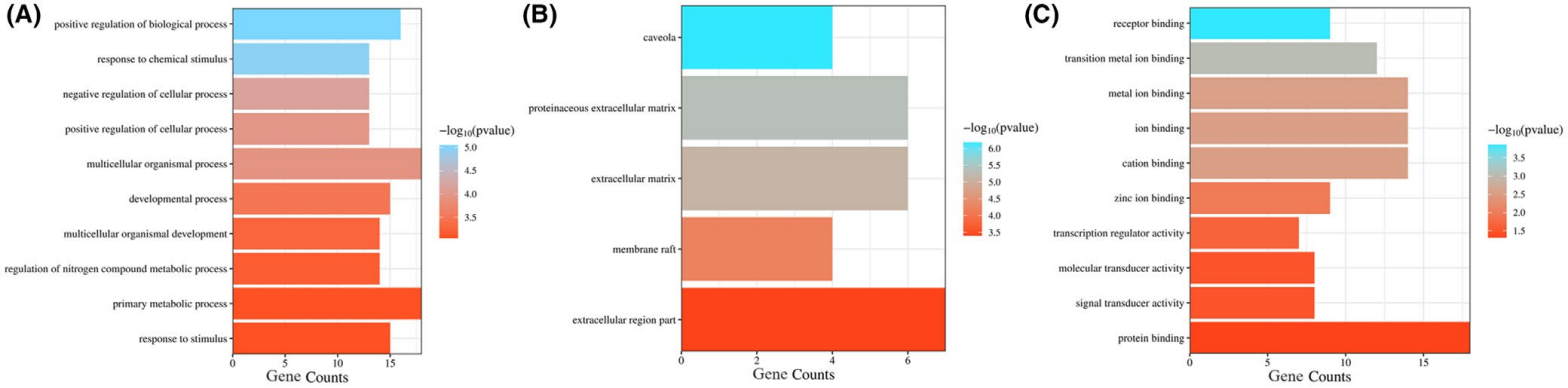
Results of GO enrichment analysis **(**A) Bar plot of the biological process category terms from GO enrichment analysis (B) Bar plot of the cellular component category terms from GO enrichment analysis (C) Bar plot of the molecular function category terms from GO enrichment analysis. BP, MF, CC (*y*‐axis), gene number (*x*‐axis) and *p*‐value (colour change). Smaller P value is indicated by red colour and larger *p* value is indicated by blue colour.

A BINGO enrichment analysis was conducted on the 25 hub genes, yielding 86 biological processes (BP) with *p* < 0.001, 5 cellular components (CC) and 81 molecular functions (MF) under the GO entry with *p* < 0.05 (Table ST3–ST5—Data S1 respectively). The top results of each category were sorted according to the p‐value from small to large and a bar plot was drawn. Figure 6 illustrates the results of the GO enrichment analysis through bar plots for the biological process category (A), the cellular component category (B) and the molecular function category (C).

Sorted by the degree of significance, the BP of these 25 genes is significantly enriched in response to stimulus (GO:50896), primary metabolic process (GO:44238), regulation of nitrogen compound metabolic process (GO:51171), multicellular organismal development (GO:7275), developmental process (GO:32502), positive regulation of cellular process (GO: 48522), response to chemical stimulus (GO: 42221), etc. Significant overrepresentation of the genes in processes such as response to stimulus, primary metabolic process and developmental process suggests that these genes play crucial roles in cellular response and development, which are essential for anti‐inflammatory activity.^43^ CC is mainly enriched in caveola (GO:5901), proteinaceous extracellular matrix (GO:5578), extracellular matrix (GO: 31012), membrane raft (GO:45121) and extracellular region part (GO:44421). Enrichment in components like caveola and extracellular matrix indicates the involvement of these genes in cell signalling and structural integrity, crucial for mediating inflammatory responses.^44^ The predominantly enriched MF terms include protein binding (GO:5515), signal transducer activity (GO:4871), transcription regulator activity (GO:30528), zinc ion binding (GO:8270), cation binding (GO:43169), receptor binding (GO:5102), metal ion binding (GO: 46872), etc. The overrepresentation of the genes in MFs such as protein binding and signal transducer activity underscores their critical roles in protein interactions and signal transduction pathways, which are essential for regulating inflammatory responses.^45^

**FIGURE 7:**
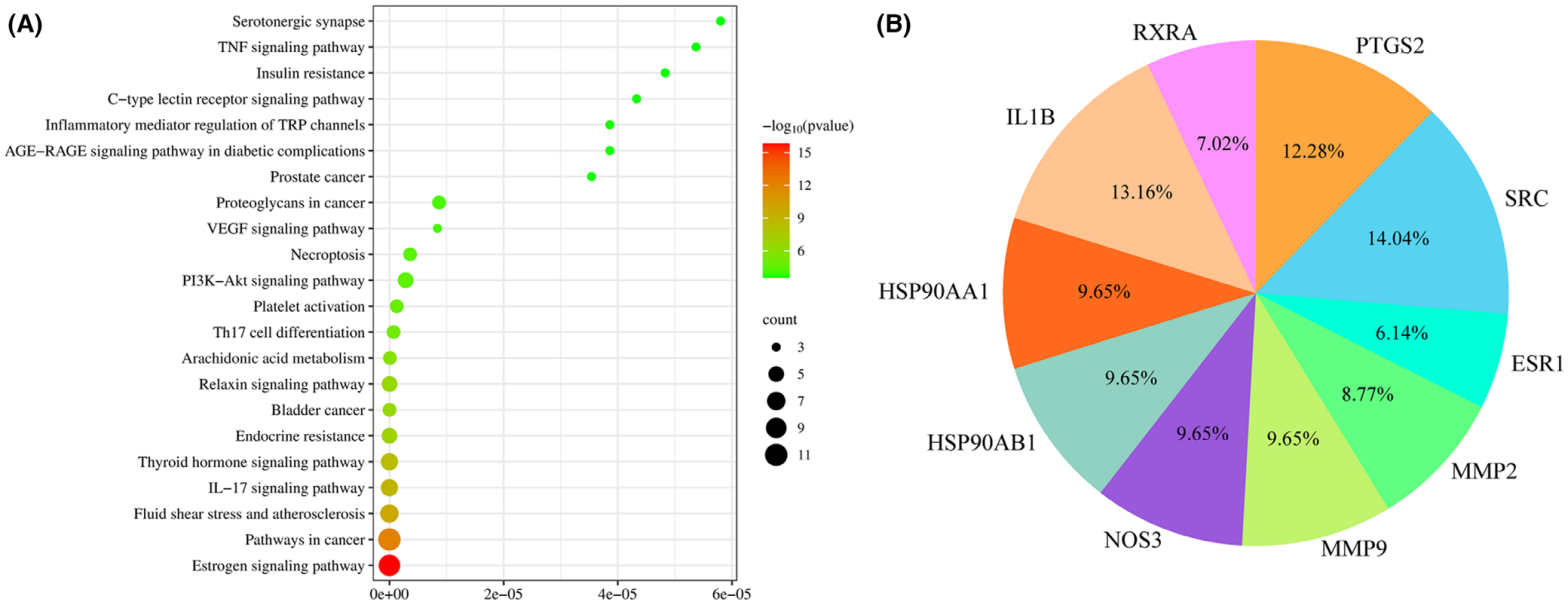
(A) Dot plot of the KEGG pathway enrichment analysis. The horizontal axis represents the enrichment rate of the input genes in the pathway, while the vertical axis represents the pathway name. The colour scale indicates different thresholds of the *p*‐value and the size of the dot indicates the number of genes corresponding to each term. (B) Pie chart illustrating the percentage of gene targets involved in various pathways.

The essential signalling pathways were elucidated through KEGG pathway enrichment analysis, utilizing the hypergeometric test/Fisher's exact test as the statistical methodology, complemented by the Benjamini and Hochberg False Discovery Rate (FDR) correction method. A total of 42 statistically significant pathways were identified with *p* < 0.001 (Table ST6—Data S1). Out of these, the top 22 signal pathways are shown in Figure 7A which were classified according to the *p*‐value from small to large, of which seven pathways are related to inflammation.

Pathway enrichment analyses indicated that the effects of *R. pectinata* are mediated by genes related to inflammation and cancer and the prominent signalling pathways identified include: Oestrogen signalling pathway, Pathways in cancer, Fluid shear stress and atherosclerosis, IL‐17 signalling pathway, etc. Among the inflammation‐related pathways, IL‐17 Signalling Pathway has a high enrichment degree and can be regarded as the main anti‐inflammatory pathway. From Figure 7B, it was observed that among the associated targets, *SRC*, *PTGS2* and *IL1B* were involved in the highest number of pathways. *IL1B*, a cytokine produced in response to inflammatory stimuli, plays a significant role in the pathogenesis of various inflammatory diseases.^46^ *PTGS2*, also known as *COX‐2*, is a protein responsible for the formation of pro‐inflammatory prostaglandins.^47^ *SRC*, a proto‐oncogene tyrosine‐protein kinase, is involved in the regulation of cell proliferation, survival and migration, with its overactivity linked to numerous cancers.^48^ Hence, *SRC*, *PTGS2* and *IL1B* were selected as the central protein targets for molecular docking analysis.

### 3.5 Construction of the herb‐compound‐target‐pathway network

**FIGURE 8:**
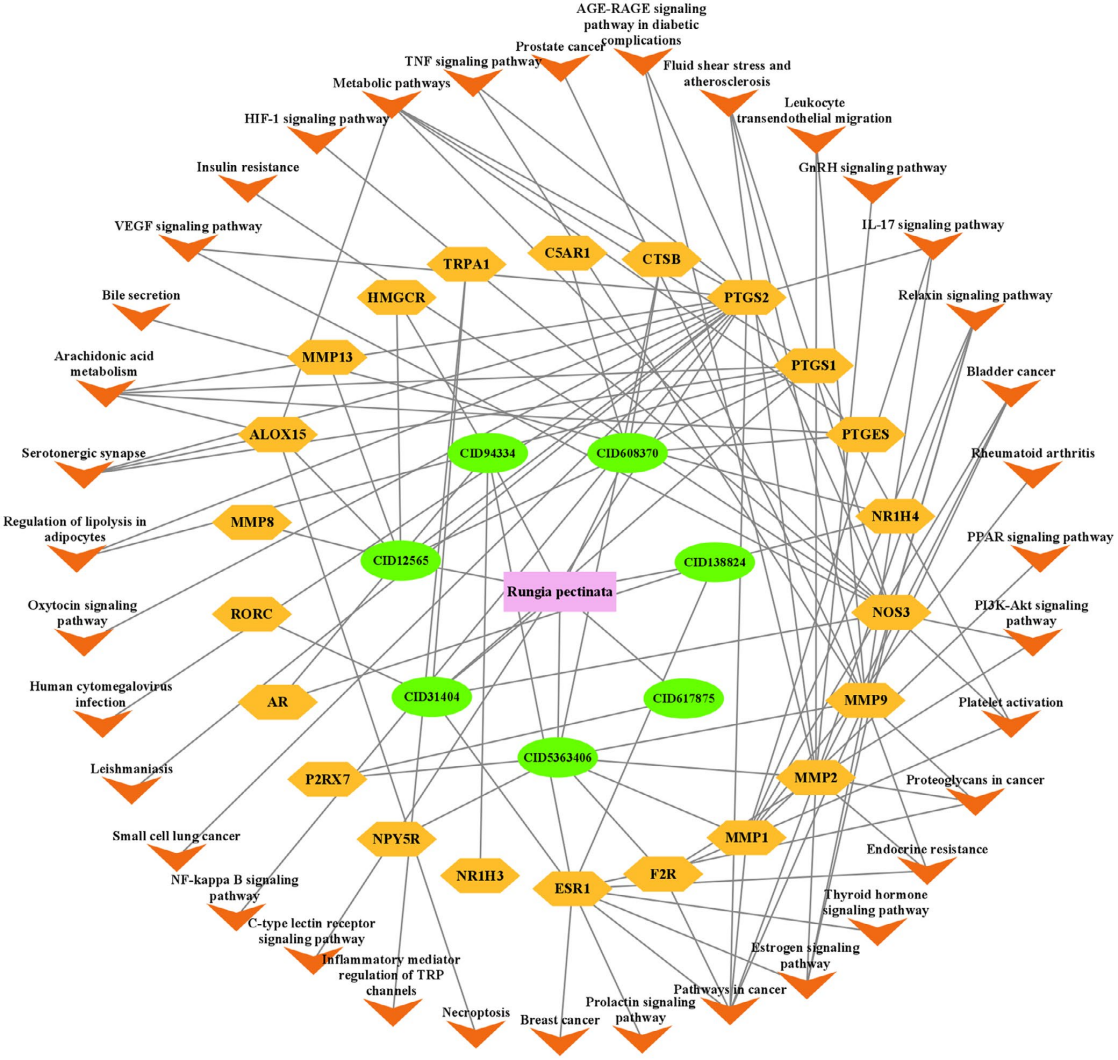
Herb‐compound‐target‐pathway network diagram. The herb is displayed as a pink rectangle, active ingredients are displayed as green ellipses, the intersection targets are displayed as yellow hexagrams and the signal pathways are displayed as orange triangles.

Using the Cytoscape 3.9.1 software, a network graph with 48 nodes and 115 edges was constructed (Figure 8).

The nodes included 7 active ingredients of *R. pectinata*, 22 potential anti‐inflammatory targets and 36 signal pathways. The ingredient attribute nodes in the network graph, CID5363406 and CID94334 have the highest degree value; these can be considered as the core nodes in the network with major anti‐inflammatory effects. The core target *PTGS2* is connected with multiple pathways, such as IL‐17 signalling pathway, arachidonic acid metabolism, VEGF signalling pathway, etc. Each active ingredient's ability to correspond to multiple targets across various pathways fully reflects the multi‐ingredient, multi‐target and multi‐pathway mechanism underlying *R. pectinata's* anti‐inflammatory effects.

### 3.6 DFT analysis

Properties like electron affinities, ionization potentials, orbital energies and molecular structures may be ascertained through DFT analysis. Table 2 displays the DFT study findings for the compounds that were chosen.

**TABLE 2:** DFT results of the seven compounds of our interest.

Ligand name	PubChem ID	E_HOMO_	E_LUMO_	Eg	Chemical Hardness (*η*)	Electronegativity (*χ*)	Chemical Potential (*μ*)	Chemical Softness (*Ѕ*)
LGA	CID608370	−0.199	−0.003	0.196	0.009	0.101	−0.101	10.190
LGB	CID12565	−0.224	0.015	0.239	0.119	0.104	−0.104	8.337
LGC	CID31404	−0.202	0.011	0.213	0.106	0.095	−0.095	9.346
LGD	CID138824	−0.267	0.069	0.336	0.168	0.099	−0.09	5.944
LGE	CID617875	−0.227	0.001	0.228	0.114	0.113	−0.113	8.738
LGF	CID5363406	**−0.177**	**−0.016**	**0.161**	**0.080**	**0.096**	**−0.096**	**12.405**
LGG	CID94334	**−0.228**	**−0.043**	**0.184**	**0.092**	**0.135**	**−0.135**	**10.814**

Of the seven compounds, LGF (CID5363406) and LGG (CID94334) had the lowest HOMO‐LUMO energy gap (δE = 0.161 and 0.184 respectively). Because the electron acceptor group has a greater ability to gain electrons, a reduced HOMO‐LUMO energy gap enhances charge transfer within the molecules.^49^ In comparison to the other molecules, LGF and LGG likewise exhibit decreased hardness and increased softness. A greater degree of softness and reactivity is associated with a smaller HOMO‐LUMO energy gap, while more stability and lower reactivity are linked to a broader gap. Additionally, compared to the other ligands, LGF and LGG have higher chemical potential (μ) and lower electronegativity values (χ), indicating higher reactivity. Based on their minimal band energy gaps, Table 2 concludes that LGF and LGG are the most reactive compounds, as indicated by the bold values in the table.

### 3.7 Prediction of binding site and molecular docking

**FIGURE 9:**
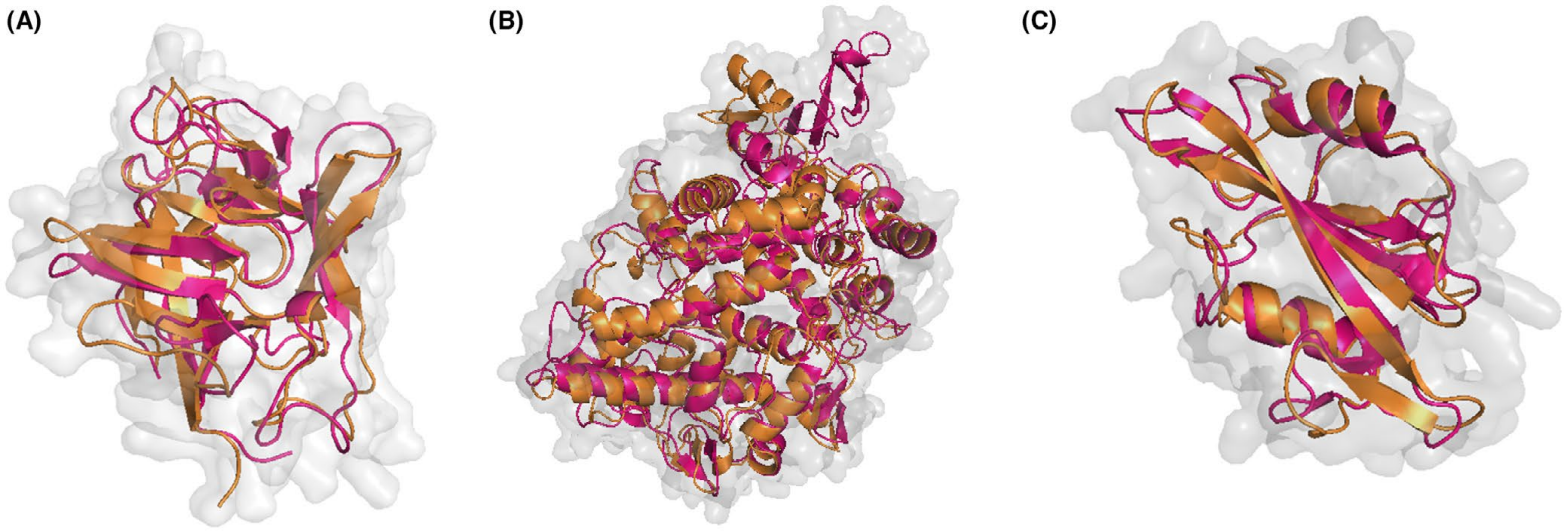
Structural superimposition of the initial (orange colour) and MD simulated (pink colour) (A) *IL1B* (B) *PTGS2* (C) *SRC*.

For this study, three central targets were identified: Interleukin‐1 beta (*IL1B*, PDB ID: 5R8Q), Prostaglandin‐endoperoxide synthase 2 (*PTGS2*, PDB ID: 5F19) and proto‐oncogene tyrosine‐protein kinase Src (*SRC*, PDB ID: 1O43) through network topological analysis. These specific PDB entries were chosen due to their high resolution (all <2.1 Å), ensuring detailed structural information, and their extensive use in previous studies, which validates their relevance and reliability.^50^, ^51^ The PDB structures of these proteins included native ligands, which allowed us to use these native ligand‐binding sites as the reference binding sites for all three proteins. However, two of the proteins, *IL1B* and *SRC*, had missing residues and atoms in their PDB structures. To address this, the complete structures of *IL1B* and *SRC* were modelled using SWISS‐MODEL. After obtaining the modelled structures, MD simulations were performed for 50 ns to refine these models further. The simulated structures of *IL1B*, *PTGS2* and *SRC* obtained from MD analysis were then used for docking studies. Figure 9 depicts the structural superimposition of the initial and MD simulated structures.

The important ligand‐binding residues in the simulated structures were compared with those in the crystal structures. The Root Mean Square Deviation (RMSD) values obtained were very small, 1.282 Å for *IL1B* and 1.118 Å for *SRC*, indicating high structural similarity. For *PTGS2*, the RMSD value was also low (1.560 Å), demonstrating that the ligand‐binding site is highly conserved. This superposition confirmed that the binding sites identified in the original PDB structures, where the native ligands were bound, could also be used as the binding sites for the simulated proteins. This approach ensured that the binding sites used for docking were accurately aligned with the native ligand‐binding sites, maintaining the structural integrity and relevance of the docking studies (Table 3). The low RMSD values for the ligand‐binding sites across all proteins indicates that the important residues superimpose well, further validating our binding site selection.^31^

**TABLE 3:** List of amino acid residues present in the binding cavity of the proteins.

Protein	Residues
*IL1B*	TYR24, GLU25, LEU26, LEU69, LEU80, GLN81, LEU82, PRO131, VAL132, PHE133
*PTGS2*	TYR148, ALA199, PHE200, ALA202, GLN203, HIS207, PHE210, LYS211, THR212, HIS214, LEU294, VAL295, ASN382, TYR385, HIS386, TRP387, HIS388, LEU390, LEU391, PHE395, TYR404, PHE407, ILE408, VAL444, VAL447, ALA450, GLN454
*SRC*	THR13, ARG34, SER36, GLU37, THR38, TYR43, CYS44, LYS59, HIS60, TYR61, LYS62, ILE73, THR74, TYR89, ASP94, GLY95, LEU96

Molecular docking was performed to identify the types of interactions and binding affinities of the studied molecules with the targets.^52^ *IL1B*, *PTGS2* and *SRC* were subjected to docking with the seven ADMET‐qualified compounds to explore the strength of their binding interactions. Among the seven compounds, two molecules, trans‐nerolidyl formate (LGF) and widdrol (LGG), demonstrated better affinity and equal or superior docking scores. Additionally, these proteins were docked with their native ligands to establish a threshold for strong interactions. The results, including docking scores, are presented in Table 4.

**TABLE 4:** CDOCKER energy and non‐covalent interactions of protein‐ligand complexes obtained from molecular docking study.

Protein	Ligand	CDOCKER Energy (kcal/mol)	H‐bonded interactions	Other non‐bonded interactions
Interleukin‐1 beta (*IL1B*)	Native	−6.488	2	7
	LGF	−32.859	2	6
	LGG	−29.541	1	7
	Native	15.397	1	12
Prostaglandin G/H synthase 2 (*PTGS2*)	LGF	−25.126	1	15
	LGG	−26.413	1	12
	Native	11.103	1	8
Proto‐oncogene tyrosine‐protein kinase Src (*SRC*)	LGF	−32.919	1	6
	LGG	−31.452	2	3

In the course of docking, the calculation of CDOCKER energy & binding energy occurred, serving as an indicator of the stability in the interaction between the ligands and the proteins.^53^ This energy assessment offers crucial insights into the strength of the bond formed at the protein's active site. In the context of our study on the anti‐inflammatory effects of *R. pectinata*, we evaluated the significance of the reported binding affinity values by comparing them with the binding energies of the native ligands. Binding affinity, measured in kcal/mol and obtained from the CDOCKER docking simulations, quantifies how strongly a ligand binds to a protein. Lower binding affinity values indicate stronger interactions, as the system releases more energy upon binding, thus forming a more stable complex. Stronger binding interactions are typically indicated by more negative binding affinity values. Generally, binding affinity values less than −5 kcal/mol represent strong, favourable interactions. Values between −5 and − 3 kcal/mol are interpreted as moderate binding interactions, while binding affinities greater than −3 kcal/mol are considered weak interactions.^38^, ^54^ The native ligands showed higher CDOCKER values compared to LGF and LGG for all the proteins, which was considered the benchmark for evaluating interaction strength. By comparing the binding affinities of LGF and LGG with those of the native ligands, it became evident that LGF and LGG exhibited superior binding energies, signifying strong interactions. This comparison underscored that the interactions of LGF and LGG with *IL1B*, *PTGS2* and *SRC* are robust, contributing to their stability within the binding cavities.

**FIGURE 10:**
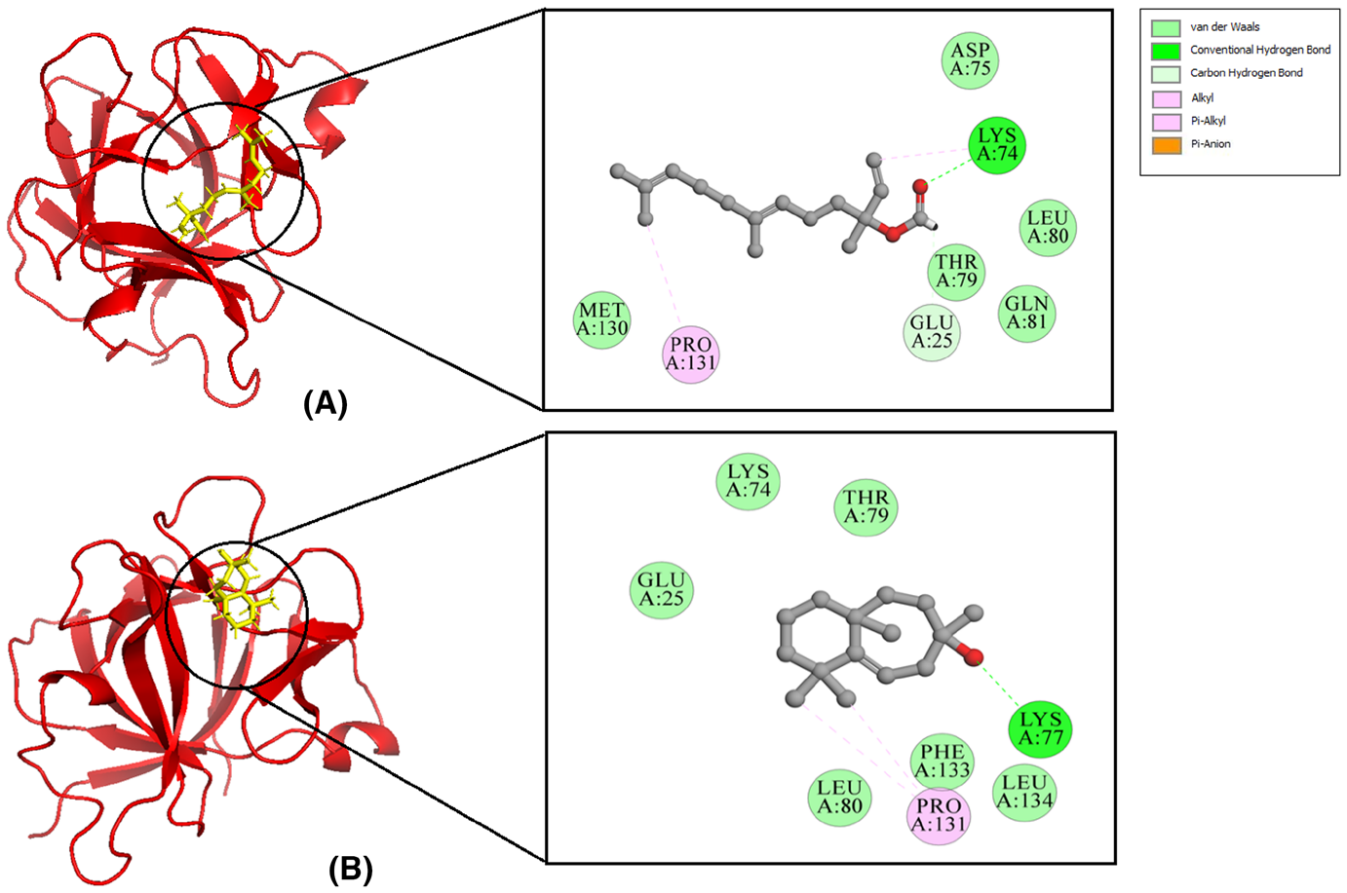
2D and 3D interaction diagram of *IL1B* with (A) LGF (B) LGG.

**FIGURE 11:**
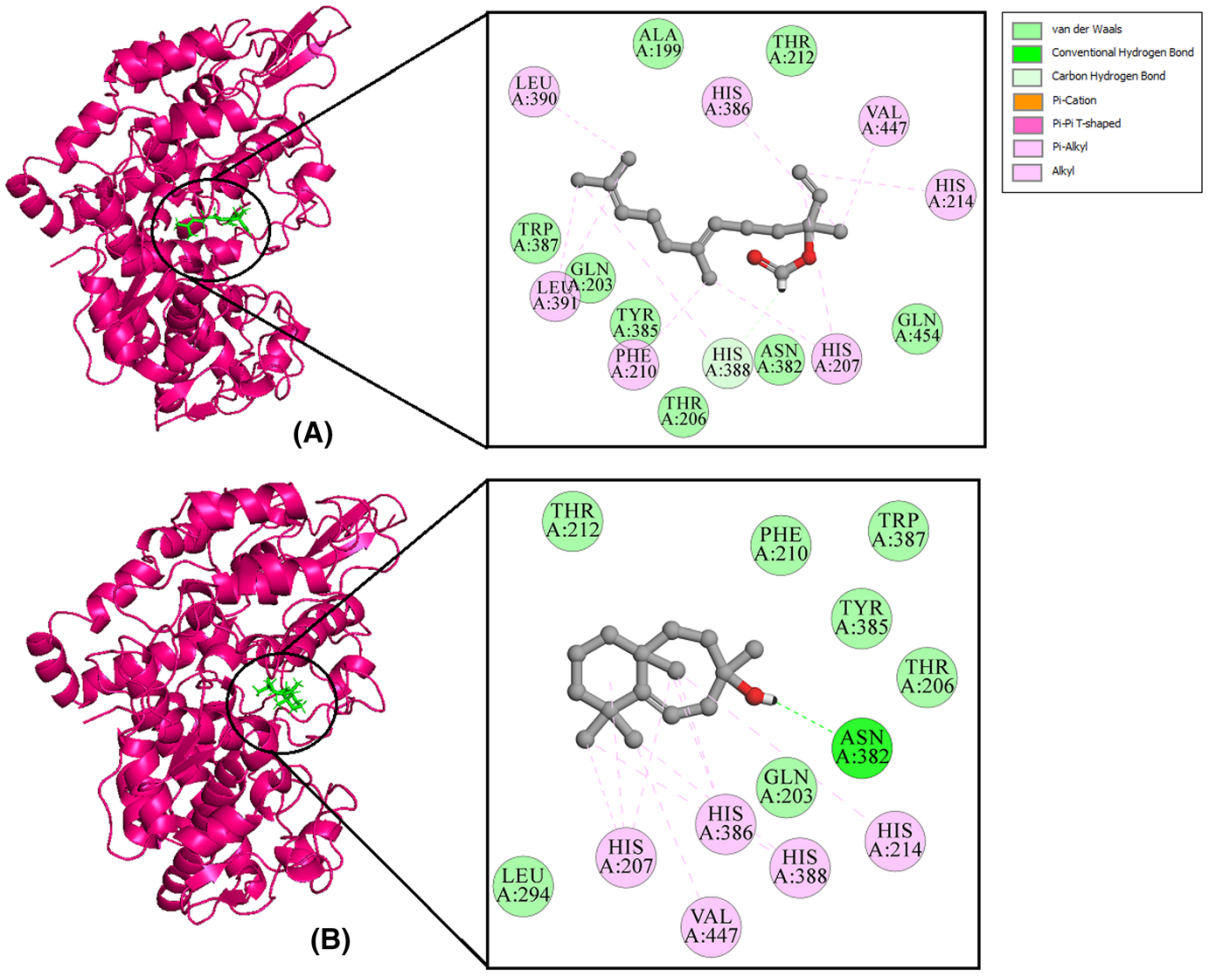
2D and 3D interaction diagram of *PTGS2* with (A) LGF (B) LGG.

**FIGURE 12:**
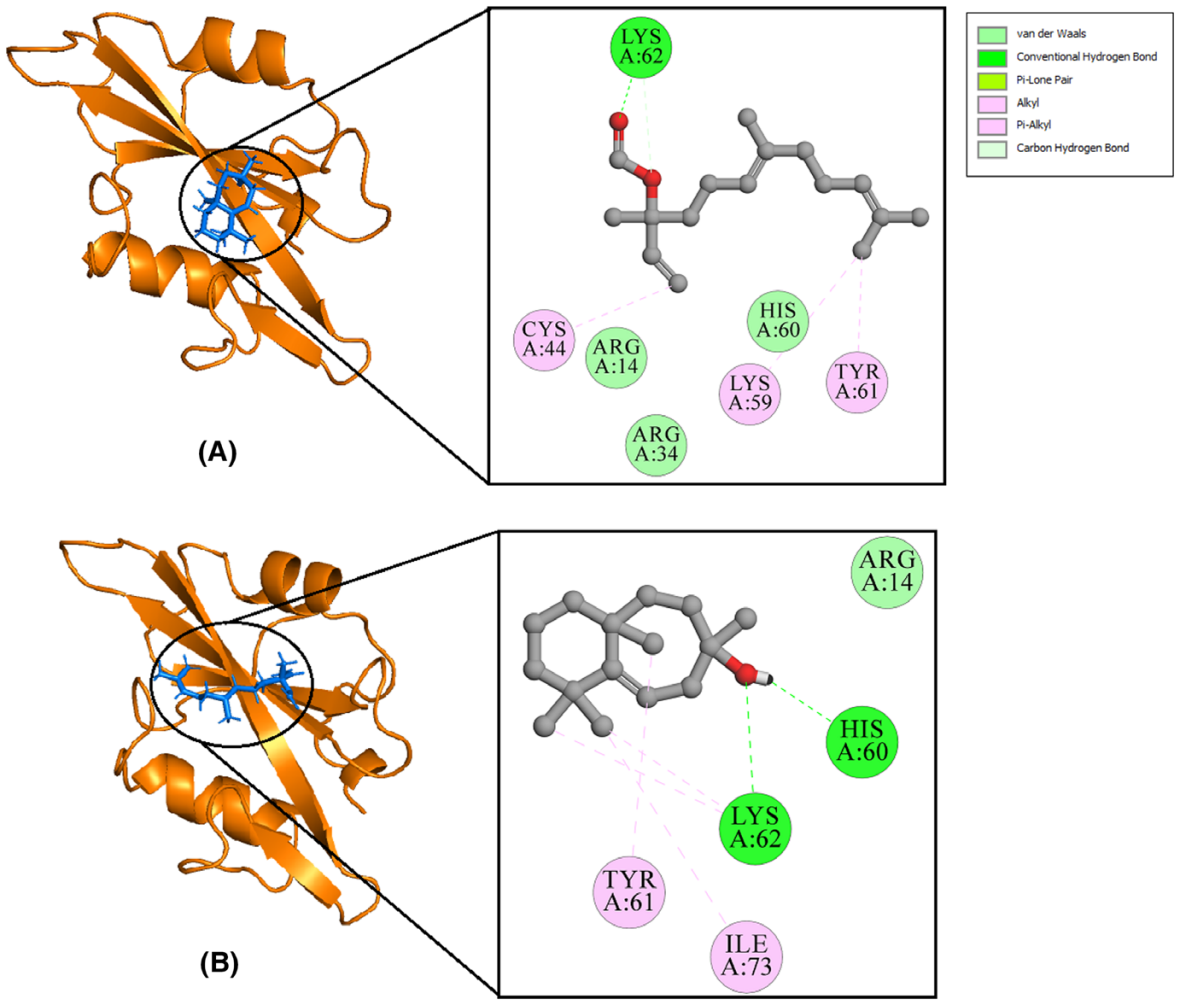
2D and 3D interaction diagram of *SRC* with (A) LGF (B) LGG.

The findings indicated that LGF and LGG successfully engaged with the target proteins, as illustrated in Figures 10, 11, 12. Moreover, an evaluation of the ligand‐protein interactions was conducted. This examination aids in comprehending the precise molecular interactions, including hydrogen bonding, hydrophobic interactions and electrostatic interactions, contributing to the stability of the ligand‐protein complex. The surrounding residues exhibited stabilization through the establishment of diverse non‐covalent interactions, including hydrogen bonds (depicted in green), Pi‐alkyl (pink), along with van der Waals interactions (mint).

From Figure 10A,B, it is observed that the LYS74 residue of the IL1B protein forms a hydrogen bond with LGF, while LYS77 forms a hydrogen bond with LGG. Figure 11A,B indicates that PTGS2 exhibits the highest number of ligand interactions, with the HIS388 residue forming a hydrogen bond with LGF and ASN382 forming a hydrogen bond with LGG. Similarly, Figure 12A,B reveals that the LYS62 residue of the SRC protein forms a hydrogen bond with LGF, and both HIS60 and LYS62 form hydrogen bonds with LGG. These interactions are all consistent with the binding patterns observed with the native ligands. Molecular docking results of all the 7 compounds are shown in Table ST7—Data S1 and Figures SF2–SF4—Data S1.

### 3.8 Analysis of MD simulations

MD simulations, each lasting 50 ns, were conducted for *IL1B*, *PTGS2* & *SRC* separately to assess the stability and fluctuations of both the protein and the docked complexes. The native proteins served as controls, and the MD simulation utilized the optimal docked position. The stability analysis was conducted using various techniques, including Root Mean Square Deviation (RMSD), Root Mean Square Fluctuation (RMSF) and the Radius of Gyration (Rg).

#### 3.8.1 Root Mean Square Deviation (RMSD)

**FIGURE 13:**
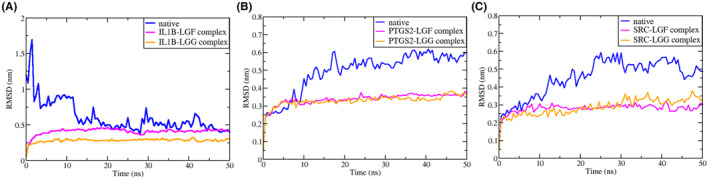
RMSD plot of (A) *IL1B* (B) *PTGS2* and (C) *SRC* in their native form and in complex with LGF and LGG at a time interval of 50 ns.

The RMSD plot, derived from MD simulations, is essential for assessing the equilibration and stability of protein structures. Smaller RMSD values suggest greater stability.^31^ RMSD values were calculated over a 50 ns simulation period for both native protein structures (*IL1B*, *PTGS2* and *SRC*) and their complexes with LGF and LGG, as shown in Figure 13.

From the RMSD plot shown in the figure, it can be observed that the RMSD of the native proteins shows a much higher rise as compared to the protein‐ligand complexes. For *IL1B* (Figure 13A), the RMSD of the native protein exhibits fluctuations up to 10 ns, stabilizing after 20 ns between 0.5 and 0.7 nm. In contrast, *IL1B* bound to LGF and LGG shows significantly more stable RMSD values, indicating enhanced protein stability. *PTGS2* (Figure 13B) displays fluctuations up to 18 ns, stabilizing after 20 ns with variations between 0.55 and 0.6 nm. In contrast, the protein‐ligand complexes of *PTGS2* demonstrate greater stability, maintaining consistent values throughout the 50 ns simulation. For *SRC* (Figure 13C), the native protein fluctuates until 24 ns, stabilizing after 30 ns with RMSD between 0.49 and 0.54 nm. On the other hand, when LGF and LGG bind to *SRC*, it demonstrates greater stability, suggesting that the ligands enhance the overall stability of the protein.

In all cases, the ligands LGF and LGG contribute to enhanced stability, as reflected in the reduced RMSD values across the simulations.

#### 3.8.2 Root Mean Square Fluctuations (RMSF)

**FIGURE 14:**
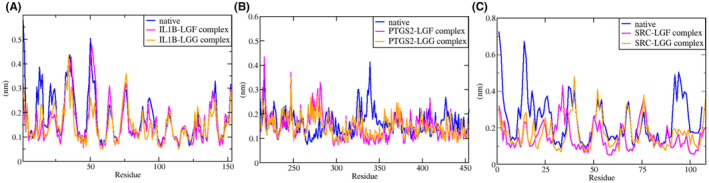
RMSF plot of (A) *IL1B* (B) *PTGS2* and (C) *SRC* in their native form and in complex with LGF and LGG at a time interval of 50 ns.

Residue‐wise fluctuations were analysed using RMSF calculations, which provide insight into the flexibility of protein residues during simulation. Understanding the flexibility of functionally important residues is essential, as changes can affect protein function. Higher RMSF values indicate greater flexibility, while lower values suggest restricted movements.^55^ Figure 14A shows the comprehensive variation of the region containing essential *IL1B* residues, while Figure 14B and Figure 14C illustrate fluctuations for *PTGS2* and *SRC* residues, respectively.

The combined Figure 14A–C indicates the overall variations in functionally critical residues of the protein‐ligand complexes compared to those observed in the native state. For *IL1B*, the graph shows that the overall fluctuations of the key residues in the complexes are similar to those in the native protein. In contrast, the RMSF plots for *PTGS2* complexes with LGF and LGG indicate generally reduced fluctuations compared to the native state. Similarly, the *SRC* complexes with LGF and LGG also exhibit reduced fluctuations relative to the native protein.

#### 3.8.3 Radius of Gyration (Rg)

**FIGURE 15:**
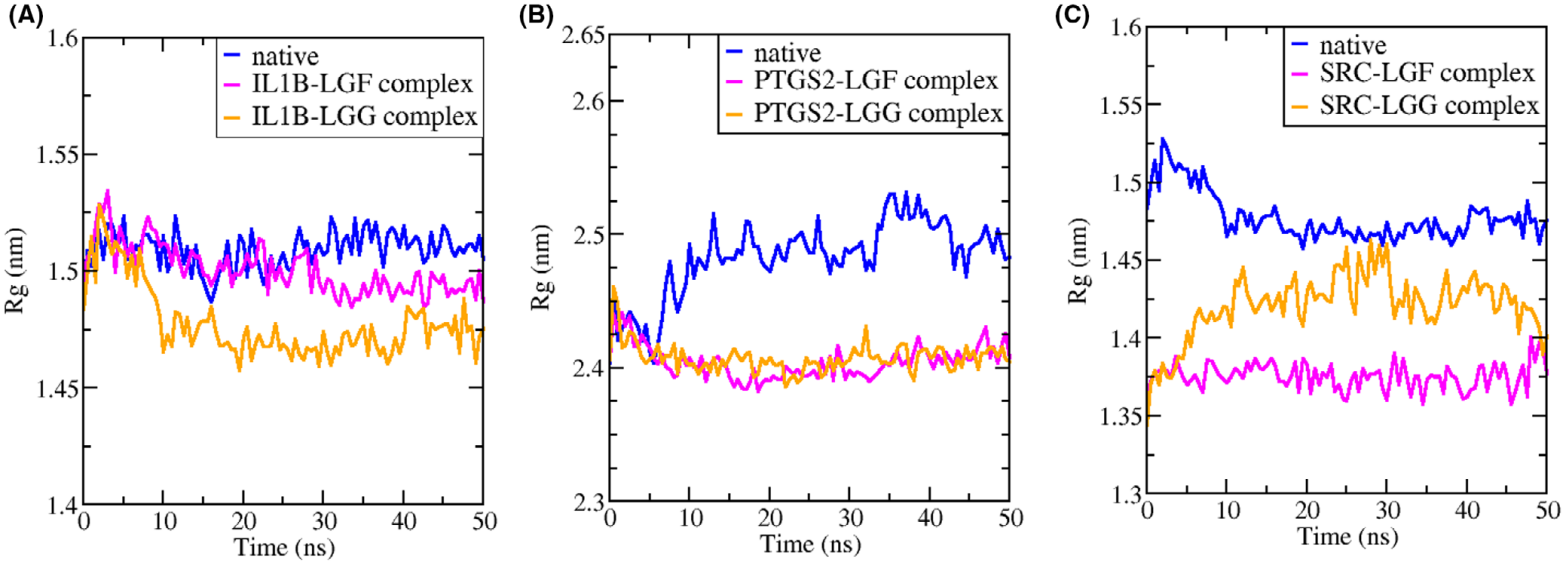
Radius of gyration plot of (A) *IL1B* (B) *PTGS2* and (C) *SRC* in their native form and in complex with LGF and LGG at a time interval of 50 ns.

The Rg plots were analysed to evaluate the compactness of the proteins and their docked complexes. Rg measures the root mean square distance of atoms from the centre of mass, providing insights into the overall dimensions and stability of the protein structure.^56^ The Rg plots for native *IL1B*, *PTGS2* and *SRC* proteins and their complexes with LGF and LGG are shown in Figure 15.

For *IL1B* (Figure 15A), the initial Rg values of the native protein are similar to the complexes, but after 25 ns, the Rg values of the complexes decrease, indicating greater compactness. Specifically, the *IL1B*‐LGF complex exhibits minimal deviations, suggesting greater compactness and stability. In the case of *PTGS2* (Figure 15B), the complexes maintain lower and more stable Rg values throughout the 50 ns simulation compared to the native protein, indicating that *PTGS2* becomes more compact when bound to LGF and LGG. For *SRC* (Figure 15C), the native protein exhibits higher Rg values throughout the 50 ns simulation while the complexes display lower and more stable values, reflecting increased compactness and stability. These results demonstrate that the binding of LGF and LGG enhances protein compactness, contributing to structural stability throughout the simulation.

#### 3.8.4 MM/PBSA analysis

Binding free energy analysis aimed to calculate the energies that were associated with the binding of the ligands to *IL1B*, *PTGS2* and *SRC* during the MD simulations. A precise estimation of binding free energies of the protein‐ligand complexes is among the most critical aspects of understanding protein‐ligand interactions.^57^ Using the MM/PBSA approach, binding energies were calculated for all complexes. The results showed that *SRC* complexes had better free energy values compared to *IL1B* and *PTGS2,* as indicated by the bold values in Table 5. van der Waals, electrostatic and SASA energies contributed significantly to binding stability, while polar solvation energy had a negative impact. Among these, van der Waals energy made the largest contribution to overall binding energy.^31^ These results highlight the strong binding affinities of the *SRC* complexes, indicating their potential relevance in cancer therapy. The binding free energy and its corresponding components, calculated using MM‐PBSA, are presented in Table 5.

**TABLE 5:** Analysis of the contribution of individual energy components in the complex formation between *IL1B*, *PTGS2*, *SRC* and the top lead molecules.

Sl. No	System	van der Waals energy (kJ/mol)	Electrostatic energy (kJ/mol)	Polar solvation energy (kJ/mol)	SASA energy (kJ/mol)	Binding free energy (kJ/mol)
1	*IL1B*‐LGG	−33.400 ± 15.266	−52.038 ± 15.314	92.779 ± 23.310	−9.756 ± 1.644	−2.414 ± 19.747
2	*IL1B*‐LGF	−29.183 ± 32.794	−7.521 ± 11.824	31.956 ± 47.843	−5.843 ± 6.373	−10.592 ± 30.437
3	*PTGS2*‐LGG	−62.790 ± 11.683	−20.402 ± 12.666	60.912 ± 19.914	−12.576 ± 1.305	−34.857 ± 12.589
4	*PTGS2*‐LGF	−65.604 ± 17.263	−16.473 ± 13.590	67.556 ± 22.157	−13.507 ± 2.782	−28.027 ± 15.244
5	*SRC*‐LGF	−82.097 ± 19.855	−18.424 ± 17.552	59.539 ± 20.714	−15.729 ± 3.039	**−56.710 ± 18.470**
6	*SRC*‐LGG	−74.870 ± 12.782	−21.098 ± 16.279	60.349 ± 22.434	−16.675 ± 4.212	**−52.293 ± 12.054**

#### 3.8.5 Hydrogen bonds and other non‐bonded interactions

Non‐covalent interactions have a major role in stabilizing the structural organization of biological entities, ranging from small chemical molecules to massive complexes in living beings and crystals. Moreover, water‐mediated solvation and the participation of ions are essential to the operation and control of biological processes. Thus, a crucial field of study in the natural sciences is comprehending the subtleties of solvation and non‐covalent interactions.^58^ Protein‐ligand stability is influenced by hydrogen bonds and non‐bonded interactions like van der Waals forces, which optimize packing and interaction energies. In MD simulations, these interactions are analysed over time to assess their impact on complex stability, offering valuable insights for drug discovery and molecular design.^59^

**FIGURE 16:**
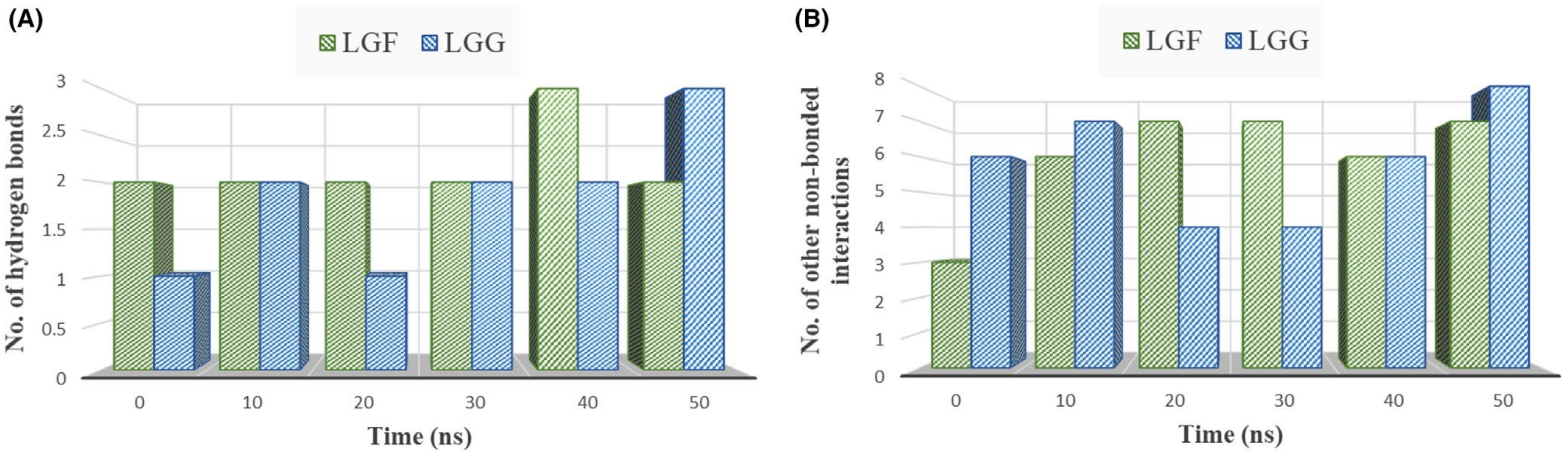
Graph showing the comparison of time evolution in number (at every 10 ns) of (A) Hydrogen bonds (B) Other non‐bonded interactions for *IL1B* in complex with LGF and LGG.

**FIGURE 17:**
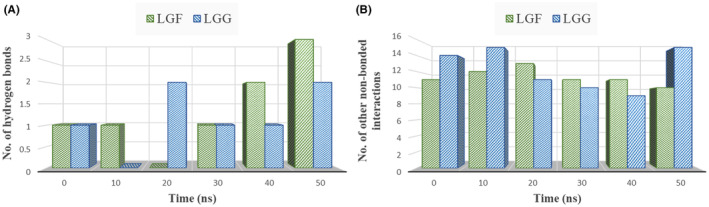
Graph showing the comparison of time evolution in number (at every 10 ns) of (A) Hydrogen bonds (B) Other non‐bonded interactions for *PTGS2* in complex with LGF and LGG.

**FIGURE 18:**
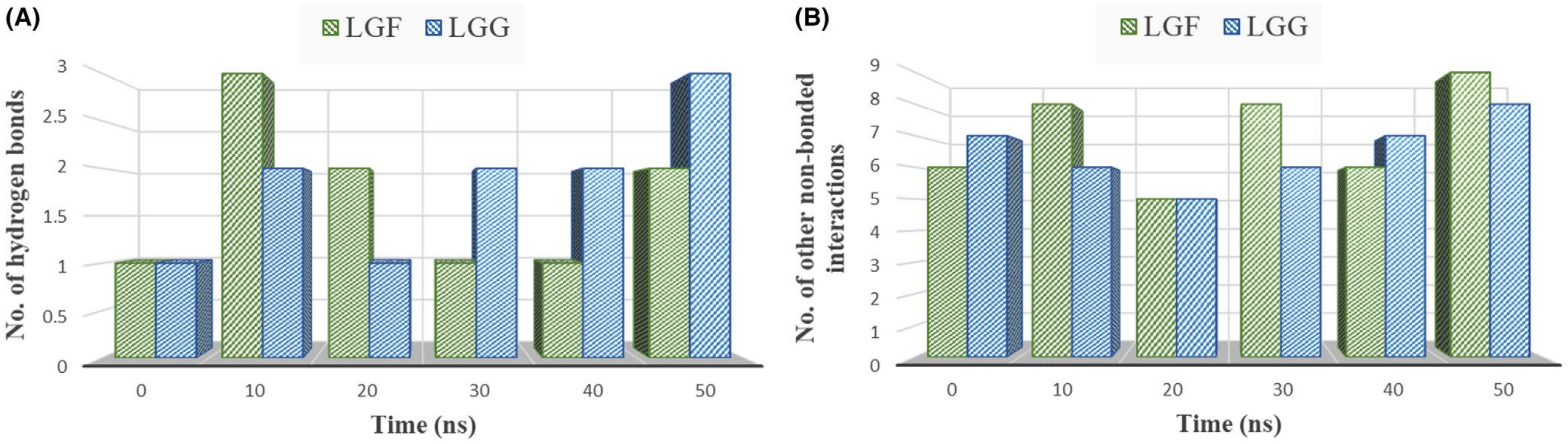
Graph showing the comparison of time evolution in number (at every 10 ns) of (A) Hydrogen bonds (B) Other non‐bonded interactions for *SRC* in complex with LGF and LGG.

In Figures 16, 17, 18, predominant hydrogen and hydrophobic interactions were observed in the initial complexes, indicating the sustained stability of the LGF and LGG compounds within the binding cavity.

The number of non‐bonded contacts between the LGF and LGG compounds and the target proteins (*IL1B*, *PTGS2*, *SRC*) at 10‐ns intervals can be noted, showing a consistent presence of hydrogen bonds (Figures 16, 17, 18) and other non‐bonded interactions (Figures 16, 17, 18) over time. This signifies a substantial contribution to the overall stability of the complexes. The increase in these interactions from the initial complex highlights the ligands' continued stability within the binding cavity.

## 4 DISCUSSION

The integrated computational approach employed in this study elucidated the anti‐inflammatory mechanisms of *Rungia pectinata* L., underscoring its potential as a therapeutic agent. Our identification of key bioactive compounds, such as trans‐nerolidyl formate and widdrol, which interact with pivotal inflammation‐related proteins including *IL1B*, *PTGS2* and *SRC*, highlights *R. pectinata's* capacity to mitigate inflammatory responses and suggests its influence on oncogenic pathways. The dual targeting of inflammatory and cancer‐related pathways by these compounds underscores their potential as multi‐targeted therapeutic agents, which may reduce the need for combination drug regimens and thereby diminish the risk of adverse drug‐target interactions.

Scientific research highlights the pivotal role of inflammation in tumour development, with chronic inflammation from persistent infections contributing to carcinogenesis. Inflammatory cells shape the tumour microenvironment, driving neoplastic growth via cell proliferation, survival and migration, which in turn leads to DNA damage, free radical production and growth factor secretion, elevating cancer risk. Anti‐inflammatory agents have shown promise in reducing cancer incidence and mortality, limiting cell migration, enhancing chemotherapy sensitivity and reducing tumour incidence.^60^ This observation highlights the broader therapeutic implications of *R. pectinata* beyond its anti‐inflammatory properties, suggesting a potential role in cancer management. The favourable binding of *R. pectinata* compounds to proto‐oncogene *SRC*, which regulates cell proliferation and metastasis, suggests a possible anti‐cancer mechanism, as *SRC* is implicated in breast, colon and lung cancers. Additionally, *IL1B* and *PTGS2* (*COX‐2*), key players in inflammation, are also linked to tumour growth, angiogenesis and metastasis across various cancer types.

To ensure that the therapeutic effects of these compounds are highly specific, minimizing potential off‐target effects, structural optimization is a critical next step to enhance their affinity and selectivity for specific protein targets. Additionally, employing advanced drug delivery systems, such as nanoparticle‐based delivery, could facilitate the targeted delivery of these compounds to inflamed tissues or tumour sites, thereby enhancing therapeutic efficacy and reducing systemic side effects. Given the intricate nature of biological networks, the modulation of key nodes such as *IL1B*, *PTGS2* and *SRC* by *R. pectinata* compounds is likely to have cascading effects across related pathways. These effects may include alterations in cytokine profiles, immune cell infiltration and angiogenesis within the tumour microenvironment, potentially contributing to both the anti‐inflammatory and anticancer activities of the herb. Future research should explore these broader network effects to provide a more comprehensive understanding of the therapeutic potential of *R. pectinata*.

These conclusions drawn are based primarily on in silico analyses. Translating these computational predictions into real‐world therapeutic applications requires rigorous in vitro and in vivo validation. The identified compounds must undergo extensive testing in relevant biological models to confirm their efficacy and safety. Furthermore, comprehensive toxicological assessments are essential to ensure that these compounds do not elicit unintended adverse effects. Given the complexity of the inflammation and cancer pathways involved, single‐target therapeutic approaches may be insufficient, highlighting the potential need for combination strategies or the development of multi‐targeted therapies.

## 5 CONCLUSION

In conclusion, this comprehensive study elucidates the multifaceted mechanisms underpinning the anti‐inflammatory and anticancer activities of *R. pectinata*. Utilizing an integrated approach of network pharmacology, molecular docking and MD simulations, we systematically identified key active ingredients and their corresponding molecular targets. The involvement of *R. pectinata* in critical inflammatory pathways, as revealed by GO and KEGG enrichment analyses, underscores its therapeutic potential in modulating inflammation and related oncogenic processes. Chronic inflammation is a well‐established contributor to carcinogenesis, promoting tumour growth through mechanisms such as cell proliferation, survival, migration, DNA damage and free radical formation. The ability of *R. pectinata* compounds to modulate these pathways positions it as a candidate for integrative cancer therapy, potentially enhancing the efficacy of existing treatments and reducing resistance. However, while this study provides critical insights into the pharmacological properties of *R. pectinata*, further experimental validation is essential to confirm the specific effects and therapeutic relevance of these compounds in clinical settings. The dual role of inflammation in immunity and cancer underscores the need for continued research to unravel the complex interplay between these processes and develop targeted therapeutic interventions. Overall, this study contributes valuable knowledge to the field of medicinal plant research, emphasizing the potential of *R. pectinata* as a natural remedy for inflammatory conditions and as a promising adjunct in cancer treatment.

## AUTHOR CONTRIBUTIONS

**Alaiha Zaheen:** Data curation (equal); formal analysis (equal); investigation (equal); methodology (equal); validation (equal); writing – original draft (equal). **Sanchaita Rajkhowa:** Conceptualization (lead); investigation (equal); methodology (equal); project administration (equal); supervision (equal); writing – review and editing (equal). **Sami A. Al‐Hussain:** Funding acquisition (equal); project administration (equal); validation (equal); writing – review and editing (equal). **Magdi E. A. Zaki:** Funding acquisition (equal); project administration (equal); supervision (equal); writing – review and editing (equal).

## CONFLICT OF INTEREST STATEMENT

The authors declare no conflicts of interest.

## Supporting information


Data S1.

## Data Availability

All data generated and/or analysed during this study are included in this published article [and its supplementary information files—Data S1].

## References

[1] Cragg GM , Newman DJ . Natural products: a continuing source of novel drug leads. Biochim Biophys Acta Gen Subj. 2013;1830(6):3670‐3695. doi:10.1016/j.bbagen.2013.02.008 PMC3672862 23428572

[2] Sakina MR , Dandiya PC , Hamdard ME , Hameed A . Preliminary psychopharmacological evaluation of *Ocimum sanctum* leaf extract. J Ethnopharmacol. 1990;28(2):143‐150. doi:10.1016/0378-8741(90)90023-M 2329804

[3] Song JX , Sun YR , Peluso I , et al. A novel curcumin analog binds to and activates TFEB in vitro and in vivo independent of MTOR inhibition. Autophagy. 2016;12(8):1372‐1389. doi:10.1080/15548627.2016.1179404 27172265 PMC4968239

[4] WHO . Traditional medicine strategy 2002‐2005. Accessed July 3, 2024 https://www.who.int/publications/i/item/WHO‐EDM‐TRM‐2002.1

[5] Pinto DCGA , Silva AMS . Anticancer natural Coumarins as Lead compounds for the discovery of new drugs. Curr Top Med Chem. 2017;17(29):3190‐3198. doi:10.2174/1568026618666171215095750 29243581

[6] Shukla S , Bajpai VK , Kim M . Plants as potential sources of natural immunomodulators. Rev Environ Sci Biotechnol. 2014;13(1):17‐33. doi:10.1007/s11157-012-9303-x

[7] Harborne AJ . Phytochemical Methods A Guide to Modern Techniques of Plant Analysis. Springer Science & Business Media; 1998.

[8] Nadkarni AK . *Dr. K. M*. Nadkarni's Indian Materia Medica: With Ayurvedic, Unani‐Tibbi, Siddha, Allopathic, Homeopathic, *Naturopathic & Home Remedies, Appendices & Indexes*. Popular Prakashan. 2007.

[9] Swain SR , Sinha BN , Murthy PN . Antiinflammatory, diuretic and antimicrobial activities of *Rungia pectinata* Linn. And *Rungia repens* Nees. Indian J Pharm Sci. 2008;70(5):679‐683. doi:10.4103/0250-474X.45418 21394276 PMC3038304

[10] Zhang Y , Gao J , Mi F , Gao P , Lai P . Chemical composition and antioxidant activity of the essential oil of the whole plant of *Rungia pectinata* . J Essent Oil Bear Plants. 2016;19(4):1043‐1046. doi:10.1080/0972060X.2016.1197800

[11] PubChem , “PubChem.” Accessed: Jun. 14, 2024. [Online]. Available: https://pubchem.ncbi.nlm.nih.gov/

[12] Pires DEV , Blundell TL , Ascher DB . pkCSM: predicting small‐molecule pharmacokinetic and toxicity properties using graph‐based signatures. J Med Chem. 2015;58(9):4066‐4072. doi:10.1021/acs.jmedchem.5b00104 25860834 PMC4434528

[13] Baruah VJ , Paul R , Gogoi D , et al. Integrated computational approach toward discovery of multi‐targeted natural products from Thumbai (*Leucas aspera*) for attuning NKT cells. J Biomol Struct Dyn. 2022;40(7):2893‐2907. doi:10.1080/07391102.2020.1844056 33179569

[14] Pantaleão SQ , Fernandes PO , Gonçalves JE , Maltarollo VG , Honorio KM . Recent advances in the prediction of pharmacokinetics properties in drug design studies: a review. ChemMedChem. 2022;17(1):e202100542. doi:10.1002/cmdc.202100542 34655454

[15] DISGENET . The most extensive gene‐disease association network. Accessed June 24, 2024. https://www.disgenet.com/

[16] Bu H , Li X , Hu L , et al. The anti‐inflammatory mechanism of the medicinal fungus puffball analysis based on network pharmacology. Inform Med Unlocked. 2021;23:100549. doi:10.1016/j.imu.2021.100549

[17] GeneCards—Human Genes|Gene Database|Gene Search. Accessed June 24, 2024. https://www.genecards.org/

[18] Safran M , Dalah I , Alexander J , et al. GeneCards version 3: the human gene integrator. Database. 2010;2010:baq020. doi:10.1093/database/baq020 20689021 PMC2938269

[19] Li R , Guo C , Li Y , Qin Z , Huang W . Therapeutic targets and signaling mechanisms of vitamin C activity against sepsis: a bioinformatics study. Brief Bioinform. 2021;22(3):bbaa079. doi:10.1093/bib/bbaa079 32393985 PMC7454291

[20] Venny 2.1.0 . Accessed June 23, 2024. https://bioinfogp.cnb.csic.es/tools/venny/

[21] von Mering C , Huynen M , Jaeggi D , Schmidt S , Bork P , Snel B . STRING: a database of predicted functional associations between proteins. Nucleic Acids Res. 2003;31(1):258‐261. 12519996 10.1093/nar/gkg034 PMC165481

[22] STRING: functional protein association networks. Accessed June 24, 2024. https://string‐db.org/

[23] Shannon P , Markiel A , Ozier O , et al. Cytoscape: a software environment for integrated models of biomolecular interaction networks. Genome Res. 2003;13(11):2498‐2504. 14597658 10.1101/gr.1239303 PMC403769

[24] Zhu N , Hou J . Molecular mechanism of the anti‐inflammatory effects of Sophorae Flavescentis Aiton identified by network pharmacology. Sci Rep. 2021;11(1):1005. doi:10.1038/s41598-020-80297-y 33441867 PMC7806711

[25] Li J , Chang RY , Chen LF , et al. Potential targets and mechanisms of Jiedu Quyu Ziyin decoction for treating SLE‐GIOP: based on network pharmacology and molecular docking. J Immunol Res. 2023;2023:e8942415. doi:10.1155/2023/8942415 PMC10072964 37026113

[26] Zhu N , Hou J , Yang N . Network pharmacology integrated with experimental validation revealed the anti‐inflammatory effects of Andrographis paniculata. Sci Rep. 2021;11(1):9752. doi:10.1038/s41598-021-89257-6 33963245 PMC8105393

[27] Wei LJ , Yang WM , Olounfeh KM , Zhao N , Wang S , Hao MF . Network pharmacology‐based identifcation of potential targets of the flower of Trollius chinensis Bunge acting on anti‐inflammatory effectss. Sci Rep. 2019;9(1):8109. doi:10.1038/s41598-019-44538-z 31147584 PMC6542797

[28] Maere S , Heymans K , Kuiper M . BiNGO: a Cytoscape plugin to assess overrepresentation of gene ontology categories in biological networks. Bioinformatics. 2005;21(16):3448‐3449. doi:10.1093/bioinformatics/bti551 15972284

[29] Bu D , Luo H , Huo P , et al. KOBAS‐i: intelligent prioritization and exploratory visualization of biological functions for gene enrichment analysis. Nucleic Acids Res. 2021;49(W1):317‐325. 10.1093/nar/gkab447 PMC8265193 34086934

[30] SRplot—Science and Research online plot . Accessed June 24, 2024. https://www.bioinformatics.com.cn/en

[31] Rajkhowa S , Pathak U , Patgiri H . Elucidating the interaction and stability of Withanone and Withaferin‐a with human serum albumin, lysozyme and hemoglobin using computational biophysical modeling. ChemistrySelect. 2022;7(12):e202103938. doi:10.1002/slct.202103938

[32] Saikia N , Jha AN , Deka RC . Interaction of pyrazinamide drug functionalized carbon and boron nitride nanotubes with pncA protein: a molecular dynamics and density functional approach. RSC Adv. 2013;3(35):15102‐15107. doi:10.1039/C3RA42534G

[33] Chattaraj PK , Maiti B , Sarkar U . Philicity: a unified treatment of chemical reactivity and selectivity. J Phys Chem A. 2003;107(25):4973‐4975. doi:10.1021/jp034707u

[34] Lee C , Yang W , Parr RG . Development of the Colle‐Salvetti correlation‐energy formula into a functional of the electron density. Phys Rev B. 1988;37(2):785‐789. 10.1103/physrevb.37.785 9944570

[35] Rajkhowa S , Borah SM , Jha AN , Deka RC . Design of Plasmodium falciparum PI(4)KIIIβ inhibitor using molecular dynamics and molecular docking methods. ChemistrySelect. 2017;2(5):1783‐1792. doi:10.1002/slct.201601052

[36] Van Der Spoel D , Lindahl E , Hess B , Groenhof G , Mark AE , Berendsen HJC . GROMACS: fast, flexible, and free. J Comput Chem. 2005;26(16):1701‐1718. doi:10.1002/jcc.20291 16211538

[37] Malde AK , Zuo L , Breeze M , et al. An automated force field topology builder (ATB) and repository: version 1.0. J Chem Theory Comput. 2011;7(12):4026‐4037. 26598349 10.1021/ct200196m

[38] Zha X , Ji R , Li Y , Cao R , Zhou S . Network pharmacology, molecular docking, and molecular dynamics simulation analysis reveal the molecular mechanism of halociline against gastric cancer. Mol Divers. 2024; 1–11. doi:10.1007/s11030-024-10822-y 38504075

[39] Lipinski CA , Lombardo F , Dominy BW , Feeney PJ . Experimental and computational approaches to estimate solubility and permeability in drug discovery and development settings. Adv Drug Deliv Rev. 1997;23(1–3):3‐25. 10.1016/s0169-409x(00)00129-0 11259830

[40] Lagunin AA , Ivanov SM , Gloriozova TA , et al. Combined network pharmacology and virtual reverse pharmacology approaches for identification of potential targets to treat vascular dementia. Sci Rep. 2020;10(1):257. doi:10.1038/s41598-019-57199-9 31937840 PMC6959222

[41] Yıldırım MA , Goh KI , Cusick ME , Barabási AL , Vidal M . Drug—target network. Nat Biotechnol. 2007;25(10):1119‐1126. doi:10.1038/nbt1338 17921997

[42] Estrada E . Virtual identification of essential proteins within the protein interaction network of yeast. Proteomics. 2006;6(1):35‐40. 16281187 10.1002/pmic.200500209

[43] Kim HP , Son KH , Chang HW , Kang SS . Anti‐inflammatory plant flavonoids and cellular action mechanisms. J Pharmacol Sci. 2004;96(3):229‐245. doi:10.1254/jphs.CRJ04003X 15539763

[44] Page MJ , Kell DB , Pretorius E . The role of lipopolysaccharide‐induced cell Signalling in chronic inflammation. Chronic Stress. 2022;6:24705470221076390. doi:10.1177/24705470221076390 35155966 PMC8829728

[45] Kuwano K , Hara N . Signal transduction pathways of apoptosis and inflammation induced by the tumor necrosis factor receptor family. Am J Respir Cell Mol Biol. 2000;22(2):147‐149. doi:10.1165/ajrcmb.22.2.f178 10657934

[46] Dinarello CA . Interleukin‐1 in the pathogenesis and treatment of inflammatory diseases. Blood. 2011;117(14):3720‐3732. doi:10.1182/blood-2010-07-273417 21304099 PMC3083294

[47] Martín‐Vázquez E , Cobo‐Vuilleumier N , López‐Noriega L , Lorenzo PI , Gauthier BR . The PTGS2/COX2‐PGE2 signaling cascade in inflammation: pro or anti? A case study with type 1 diabetes mellitus. Int J Biol Sci. 2023;19(13):4157‐4165. doi:10.7150/ijbs.86492 37705740 PMC10496497

[48] De Kock L , Freson K . The (Patho)biology of SRC kinase in platelets and megakaryocytes. Medicina. 2020;56(12):633. doi:10.3390/medicina56120633 33255186 PMC7759910

[49] Saikia N , Seel M , Pandey R . Stability and electronic properties of 2D nanomaterials conjugated with pyrazinamide chemotherapeutic: a first‐principles cluster study. J Phys Chem C. 2016;120(36):20323. doi:10.1021/acs.jpcc.6b06000

[50] Periyasami G , Antonisamy P , Perumal K , Stalin A , Rahaman M , Alothman AA . A competent synthesis and efficient anti‐inflammatory responses of isatinimino acridinedione moiety *via* suppression of *in vivo* NF‐κB, COX‐2 and iNOS signaling. Bioorg Chem. 2019;90:103047. doi:10.1016/j.bioorg.2019.103047 31234130

[51] Gao M , Xue X , Zhang X , et al. Discovery of potential active ingredients of Er‐Zhi‐wan, a famous traditional Chinese formulation, in model rat serum for treating osteoporosis with kidney‐yin deficiency by UPLC‐Q/TOF‐MS and molecular docking. J Chromatogr B. 2022;1208:123397. doi:10.1016/j.jchromb.2022.123397 35921699

[52] Das A , Rajkhowa S , Sinha S , Zaki MEA . Unveiling potential repurposed drug candidates for *plasmodium falciparum* through in silico evaluation: a synergy of structure‐based approaches, structure prediction, and molecular dynamics simulations. Comput Biol Chem. 2024;110:108048. doi:10.1016/j.compbiolchem.2024.108048 38471353

[53] Rajkhowa S , Deka RC . Protein‐Ligand Docking Methodologies and Its Application in Drug Discovery. Oncology: Breakthroughs in Research and Practice. IGI Global; 2017:891‐914. doi:10.4018/978-1-5225-0549-5.ch035

[54] Song Y , Sakharkar MK , Yang J . Probing the mechanism of action (MOA) of Solanum nigrum Linn against breast cancer using network pharmacology and molecular docking. SN Appl Sci. 2023;5(5):133. doi:10.1007/s42452-023-05356-1

[55] Bora N , Nath JA . An integrative approach using systems biology, mutational analysis with molecular dynamics simulation to challenge the functionality of a target protein. Chem Biol Drug Des. 2019;93(6):1050‐1060. doi:10.1111/cbdd.13502 30891955

[56] Khataniar A , Das A , Baruah MJ , et al. An integrative approach to study the inhibition of Providencia vermicola FabD using C2‐quaternary Indolinones. Drug Des Devel Ther. 2023;17:3325‐3347. 10.2147/DDDT.S427193 PMC10657194 38024529

[57] Jha RK , Khan RJ , Amera GM , et al. Identification of promising molecules against MurD ligase from Acinetobacter baumannii: insights from comparative protein modelling, virtual screening, molecular dynamics simulations and MM/PBSA analysis. J Mol Model. 2020;26(11):304. doi:10.1007/s00894-020-04557-4 33068184

[58] Mahadevi AS , Sastry GN . Cation−π interaction: its role and relevance in chemistry, biology, and material science. Chem Rev. 2013;113(3):2100‐2138. doi:10.1021/cr300222d 23145968

[59] Molecular Simulations of Noncovalent Interactions in Complex Biological Systems—ProQuest. Accessed July 3, 2024. https://www.proquest.com/openview/24e8e861d4d80cb6ab9cdb391d1a9a12/1?pq‐origsite=gscholar&cbl=18750&diss=y

[60] Khataniar A , Rajkhowa S , Das A , Zaki ME . Targeting Inflammatory Proteins for Inhibition of Cell Proliferation in Tumor Microenvironment. Springer; 2024 Accessed May 29, 2024. 10.1007/16833_2024_279

