# Supplementary material for: Integrated computational strategies for Polypharmacological profiling and identification of anti‐inflammatory targets in Rungia pectinata L
Source: J Cell Mol Med. 2024 Dec 4;28(23):e70158. doi: 10.1111/jcmm.70158 (PMC11615512; doi:10.1111/jcmm.70158)
Supplement: Supplementary file 1 — Data S1. [file JCMM-28-e70158-s001.docx]

**Integrated Computational Strategies for Polypharmacological Profiling and Identification of Anti-Inflammatory Targets in of *Rungia pectinata* L.**

Alaiha Zaheen, ^1^ Sanchaita Rajkhowa, ^1*^ Sami A. Al-Hussain,^2^ and Magdi E. A. Zaki,^2*^

^1^Centre for Biotechnology and Bioinformatics, Dibrugarh University, Dibrugarh-786004, Assam, India.

^2^ Department of Chemistry, Imam Mohammad Ibn Saud Islamic University (IMSIU), Riyadh, Saudi Arabia.

^*^Correspondence: (Dr. Sanchaita Rajkhowa) [s_rajkhowa@dibru.ac.in](mailto:s_rajkhowa@dibru.ac.in) (ORCID ID: 0000-0002-4834-2654) and (Dr. Magdi E. A. Zaki) [mezaki@imamu.edu.sa](mailto:mezaki@imamu.edu.sa)

**Supplementary Information**

**Identification of chemical compound descriptors**

Chemical and biological activities of the ligands can be understood by studying these descriptors. From the highest occupied molecular orbitals (HOMO)-lowest unoccupied molecular orbitals (LUMO) energy, the ionization energy (IE), electron affinity (EA),electronegativity (χ), electronic chemical potential (μ), chemical hardness (η), chemical softness (σ) and electrophilicity index (ω) may be calculated, using the following equations:

Ionization Energy (IE) = -εHOMO

Electron Affinity (EA) = -εLUMO

Electronegativity (χ) = (IE+EA)/2

Electronic Chemical Potential (μ) = -χ

Chemical Hardness (η) = (IE-EA)/2

Chemical Softness (σ) = 1/η

Electrophilicity index (ω) = μ/2

**Results**

**Table ST1: Target prediction results of SwissTargetPrediction**

| **Compound** | **Sl. No:** | **Uniprot ID** | **Target Name** | **Target class** |  |
| --- | --- | --- | --- | --- | --- |
| Dibunol (CID31404) | 1 | P00918 | Carbonic anhydrase II | Lyase | CA2 |
|  | 2 | P14867 P47870 P18507 | GABA-A receptor; alpha-1/beta-2/gamma-2 | Ligand-gated ion channel | GABRA1 GABRB2 GABRG2 |
|  | 3 | P41595 | Serotonin 2b (5-HT2b) receptor | Family A G protein-coupled receptor | HTR2B |
|  | 4 | P28472 P18507 P14867 | GABA-A receptor; alpha-1/beta-3/gamma-2 | Ligand-gated ion channel | GABRB3 GABRG2 GABRA1 |
|  | 5 | P23219 | Cyclooxygenase-1 | Oxidoreductase | PTGS1 |
|  | 6 | P23975 | Norepinephrine transporter | Electrochemical transporter | SLC6A2 |
|  | 7 | P28335 | Serotonin 2c (5-HT2c) receptor | Family A G protein-coupled receptor | HTR2C |
|  | 8 | O75899 Q9UBS5 | GABA-B receptor | Family C G protein-coupled receptor | GABBR2 GABBR1 |
|  | 9 | P51449 | Nuclear receptor ROR-gamma | Nuclear receptor | RORC |
|  | 10 | P62508 | Estrogen-related receptor gamma | Nuclear receptor | ESRRG |
|  | 11 | O75762 | Transient receptor potential cation channel subfamily A member 1 | Voltage-gated ion channel | TRPA1 |
|  | 12 | Q92731 | Estrogen receptor beta | Nuclear receptor | ESR2 |
|  | 13 | P03372 | Estrogen receptor alpha | Nuclear receptor | ESR1 |
|  | 14 | P50406 | Serotonin 6 (5-HT6) receptor | Family A G protein-coupled receptor | HTR6 |
|  | 15 | P14679 | Tyrosinase | Oxidoreductase | TYR |
|  | 16 | P29475 | Nitric-oxide synthase, brain | Enzyme | NOS1 |
|  | 17 | P29474 | Nitric-oxide synthase, endothelial | Enzyme | NOS3 |
|  | 18 | P04278 | Testis-specific androgen-binding protein | Secreted protein | SHBG |
|  | 19 | Q05469 | Hormone sensitive lipase | Enzyme | LIPE |
|  | 20 | P00918 | Carbonic anhydrase II | Lyase | CA2 |
| Ageratochromene (CID12565) | 1 | P11926 | Ornithine decarboxylase | Lyase | ODC1 |
|  | 2 | P04035 | HMG-CoA reductase | Oxidoreductase | HMGCR |
|  | 3 | P16050 | Arachidonate 15-lipoxygenase | Enzyme | ALOX15 |
|  | 4 | P23219 | Cyclooxygenase-1 | Oxidoreductase | PTGS1 |
|  | 5 | P35354 | Cyclooxygenase-2 | Oxidoreductase | PTGS2 |
|  | 6 | P18054 | Arachidonate 12-lipoxygenase | Enzyme | ALOX12 |
|  | 7 | P49798 | Regulator of G-protein signaling 4 | Unclassified protein | RGS4 |
|  | 8 | Q99685 | Monoglyceride lipase | Enzyme | MGLL |
|  | 9 | P45452 | Matrix metalloproteinase 13 | Protease | MMP13 |
|  | 10 | P22894 | Matrix metalloproteinase 8 | Protease | MMP8 |
|  | 11 | Q14833 | Metabotropic glutamate receptor 4 | Family C G protein-coupled receptor | GRM4 |
|  | 12 | Q14416 | Metabotropic glutamate receptor 2 (by homology) | Family C G protein-coupled receptor | GRM2 |
|  | 13 | P24557 | Thromboxane-A synthase | Cytochrome P450 | TBXAS1 |
|  | 14 | P15538 | Cytochrome P450 11B1 | Cytochrome P450 | CYP11B1 |
|  | 15 | P25021 | Histamine H2 receptor | Family A G protein-coupled receptor | HRH2 |
|  |  | P19099 | Cytochrome P450 11B2 | Cytochrome P450 | CYP11B2 |
|  |  | Q07343 | Phosphodiesterase 4B | Phosphodiesterase | PDE4B |
|  |  | Q13946 | Phosphodiesterase 7A | Phosphodiesterase | PDE7A |
|  |  | O00311 | **Cell division cycle 7-related protein kinase** | Kinase | CDC7 |
|  |  | Q99558 | Mitogen-activated protein kinase kinasekinase 14 | Kinase | MAP3K14 |
| Widdrol (CID94334) | 1 | P05093 | Cytochrome P450 17A1 | Cytochrome P450 | CYP17A1 |
|  | 2 | P11511 | Cytochrome P450 19A1 | Cytochrome P450 | CYP19A1 |
|  | 3 | Q13133 | LXR-alpha | Nuclear receptor | NR1H3 |
|  | 4 | P51449 | Nuclear receptor ROR-gamma | Nuclear receptor | RORC |
|  | 5 | Q12772 | Sterol regulatory element-binding protein 2 | Unclassified protein | SREBF2 |
|  | 6 | Q9UHC9 | Niemann-Pick C1-like protein 1 | Other membrane protein | NPC1L1 |
|  | 7 | Q16850 | Cytochrome P450 51 (by homology) | Cytochrome P450 | CYP51A1 |
|  | 8 | P04035 | HMG-CoA reductase | Oxidoreductase | HMGCR |
|  | 9 | P10275 | Androgen Receptor | Nuclear receptor | AR |
|  | 10 | P03372 | Estrogen receptor alpha | Nuclear receptor | ESR1 |
|  | 11 | P04278 | Testis-specific androgen-binding protein | Secreted protein | SHBG |
|  | 12 | P06276 | Butyrylcholinesterase | Hydrolase | BCHE |
|  | 13 | Q92731 | Estrogen receptor beta | Nuclear receptor | ESR2 |
|  | 14 | P35398 | Nuclear receptor ROR-alpha | Nuclear receptor | RORA |
|  | 15 | Q14994 | Nuclear receptor subfamily 1 group I member 3 (by homology) | Nuclear receptor | NR1I3 |
|  |  | P11413 | Glucose-6-phosphate 1-dehydrogenase | Enzyme | G6PD |
|  |  | P16662 | UDP-glucuronosyltransferase 2B7 | Enzyme | UGT2B7 |
|  |  | P08172 | Muscarinic acetylcholine receptor M2 | Family A G protein-coupled receptor | CHRM2 |
|  |  | P22303 | Acetylcholinesterase | Hydrolase | ACHE |
|  |  | P23975 | Norepinephrine transporter | Electrochemical transporter | SLC6A2 |
| 7-t-Butyl-3,3-dimethyl-1-indanone (CID608370) | 1 | P22303 | Acetylcholinesterase | Hydrolase | ACHE |
|  | 2 | P07327 | Alcohol dehydrogenase alpha chain | Oxidoreductase | ADH1A |
|  | 3 | Q07973 | Cytochrome P450 24A1 (by homology) | Enzyme | CYP24A1 |
|  | 4 | P06276 | Butyrylcholinesterase | Hydrolase | BCHE |
|  | 5 | O14684 | Prostaglandin E synthase | Enzyme | PTGES |
|  | 6 | Q9NR63 | Cytochrome P450 26B1 | Cytochrome P450 | CYP26B1 |
|  | 7 | O43174 | Cytochrome P450 26A1 | Cytochrome P450 | CYP26A1 |
|  | 8 | P41594 | Metabotropic glutamate receptor 5 | Family C G protein-coupled receptor | GRM5 |
|  | 9 | P14061 | Estradiol 17-beta-dehydrogenase 1 | Enzyme | HSD17B1 |
|  | 10 | P23141 | Acyl coenzyme A:cholesterol acyltransferase | Enzyme | CES1 |
|  | 11 | P28845 | 11-beta-hydroxysteroid dehydrogenase 1 | Enzyme | HSD11B1 |
|  | 12 | P43235 | Cathepsin K | Protease | CTSK |
|  | 13 | P07858 | Cathepsin (B and K) | Protease | CTSB |
|  | 14 | P21730 | C5a anaphylatoxin chemotactic receptor | Family A G protein-coupled receptor | C5AR1 |
|  | 15 | P06401 | Progesterone receptor | Nuclear receptor | PGR |
|  |  | P11940 | Polyadenylate-binding protein 1 | Unclassified protein | PABPC1 |
|  |  | P49286 | Melatonin receptor 1B | Family A G protein-coupled receptor | MTNR1B |
|  |  | P00326 | Alcohol dehydrogenase gamma chain | Oxidoreductase | ADH1C |
|  |  | P28702 | Retinoid X receptor beta | Nuclear receptor | RXRB |
|  |  | P48443 | Retinoid X receptor gamma | Nuclear receptor | RXRG |
| Hexa-hydro-farnesol (CID138824) | 1 | P30305 | Dual specificity phosphatase Cdc25B | Phosphatase | CDC25B |
|  | 2 | P30304 | Dual specificity phosphatase Cdc25A | Phosphatase | CDC25A |
|  | 3 | Q7Z2W7 | Transient receptor potential cation channel subfamily M member 8 | Voltage-gated ion channel | TRPM8 |
|  | 4 | P00918 | Carbonic anhydrase II | Lyase | CA2 |
|  | 5 | P22748 | Carbonic anhydrase IV | Lyase | CA4 |
|  | 6 | Q14994 | Nuclear receptor subfamily 1 group I member 3 | Nuclear receptor | NR1I3 |
|  | 7 | P10275 | Androgen Receptor | Nuclear receptor | AR |
|  | 8 | P03372 | Estrogen receptor alpha | Nuclear receptor | ESR1 |
|  | 9 | Q92731 | Estrogen receptor beta | Nuclear receptor | ESR2 |
|  | 10 | P00915 | Carbonic anhydrase I | Lyase | CA1 |
|  | 11 | P16662 | UDP-glucuronosyltransferase 2B7 | Enzyme | UGT2B7 |
|  | 12 | P04278 | Testis-specific androgen-binding protein | Secreted protein | SHBG |
|  | 13 | P28845 | 11-beta-hydroxysteroid dehydrogenase 1 | Enzyme | HSD11B1 |
|  | 14 | Q15465 | Sonic hedgehog protein (by homology) | Unclassified protein | SHH |
|  | 15 | Q96RI1 | Bile acid receptor FXR | Nuclear receptor | NR1H4 |
|  |  | P21554 | Cannabinoid receptor 1 (by homology) | Family A G protein-coupled receptor | CNR1 |
|  |  | P34972 | Cannabinoid receptor 2 | Family A G protein-coupled receptor | CNR2 |
|  |  | P17252 | Protein kinase C alpha | Kinase | PRKCA |
|  |  | Q9UHC9 | Niemann-Pick C1-like protein 1 | Other membrane protein | NPC1L1 |
|  |  | P05093 | Cytochrome P450 17A1 | Cytochrome P450 | CYP17A1 |
| 3,3,4,5,5,8-hexamethyl-6,7-dihydro-2*H*-s-indacen-1-one (CID617875) | 1 | P22303 | Acetylcholinesterase | Hydrolase | ACHE |
|  | 2 | P04818 | Thymidylate synthase | Transferase | TYMS |
|  | 3 | Q99572 | P2X purinoceptor 7 | Ligand-gated ion channel | P2RX7 |
|  | 4 | P41594 | Metabotropic glutamate receptor 5 | Family C G protein-coupled receptor | GRM5 |
|  | 5 | P43235 | Cathepsin K | Protease | CTSK |
|  | 6 | P06401 | Progesterone receptor | Nuclear receptor | PGR |
|  | 7 | P10070 | Zinc finger protein GLI2 | Transcription factor | GLI2 |
|  | 8 | P08151 | Zinc finger protein GLI1 | Transcription factor | GLI1 |
|  | 9 | P30536 | Translocator protein (by homology) | Membrane receptor | TSPO |
|  | 10 | P05093 | Cytochrome P450 17A1 | Cytochrome P450 | CYP17A1 |
|  | 11 | P11309 | Serine/threonine-protein kinase PIM1 | Kinase | PIM1 |
|  | 12 | P49286 | Melatonin receptor 1B | Family A G protein-coupled receptor | MTNR1B |
|  | 13 | P07327 | Alcohol dehydrogenase alpha chain | Oxidoreductase | ADH1A |
|  | 14 | P48443 | Retinoid X receptor gamma (by homology) | Nuclear receptor | RXRG |
|  | 15 | P19793 | Retinoid X receptor alpha | Nuclear receptor | RXRA |
|  |  | P28702 | Retinoid X receptor beta | Nuclear receptor | RXRB |
|  |  | P13631 | Retinoic acid receptor gamma | Nuclear receptor | RARG |
|  |  | P10826 | Retinoic acid receptor beta | Nuclear receptor | RARB |
|  |  | P10276 | Retinoic acid receptor alpha | Nuclear receptor | RARA |
|  |  | Q9NR63 | Cytochrome P450 26B1 | Cytochrome P450 | CYP26B1 |
| trans-Nerolidylformate (CID5363406) | 1 | P20309 | Muscarinic acetylcholine receptor M3 | Family A G protein-coupled receptor | CHRM3 |
|  | 2 | P17948 | Vascular endothelial growth factor receptor 1 | Kinase | FLT1 |
|  | 3 | P35968 | Vascular endothelial growth factor receptor 2 | Kinase | KDR |
|  | 4 | Q15761 | Neuropeptide Y receptor type 5 | Family A G protein-coupled receptor | NPY5R |
|  | 5 | P52732 | Kinesin-like protein 1 | Other cytosolic protein | KIF11 |
|  | 6 | P14780 | Matrix metalloproteinase 9 | Protease | MMP9 |
|  | 7 | P03956 | Matrix metalloproteinase 1 | Protease | MMP1 |
|  | 8 | P08253 | Matrix metalloproteinase 2 | Protease | MMP2 |
|  | 9 | P25116 | Proteinase-activated receptor 1 | Family A G protein-coupled receptor | F2R |
|  | 10 | P43235 | Cathepsin K | Protease | CTSK |
|  |  | P07711 | Cathepsin L | Protease | CTSL |
|  |  | P07858 | Cathepsin (B and K) | Protease | CTSB |
|  |  | Q99572 | P2X purinoceptor 7 | Ligand-gated ion channel | P2RX7 |
|  |  | P28223 | Serotonin 2a (5-HT2a) receptor | Family A G protein-coupled receptor | HTR2A |
|  |  | P28335 | Serotonin 2c (5-HT2c) receptor | Family A G protein-coupled receptor | HTR2C |
|  |  | P55072 | Transitional endoplasmic reticulum ATPase | Primary active transporter | VCP |
|  |  | P53350 | Serine/threonine-protein kinase PLK1 | Kinase | PLK1 |
|  |  | P07099 | Epoxide hydrolase 1 | Protease | EPHX1 |
|  |  | P34913 | Epoxide hydratase | Protease | EPHX2 |
|  |  | P30536 | Translocator protein (by homology) | Membrane receptor | TSPO |

**Table ST2: Top 25 genes involved in the Protein interaction network (PIN)**

| **Gene name** | **Degree** | **Closeness** | **Betweenness** | **Radiality** |
| --- | --- | --- | --- | --- |
| TIMP1 | 19 | 53.6119 | 3516.43 | 5.41349 |
| NCOA1 | 18 | 49.6 | 4102.863 | 5.12717 |
| PTGS2 | 17 | 47.52143 | 3660 | 4.9575 |
| SREBF1 | 17 | 41.51587 | 2509.153 | 4.16749 |
| MMP9 | 16 | 50.44524 | 914.6416 | 5.34456 |
| RXRA | 15 | 46.18214 | 1871.352 | 4.73481 |
| HSP90AA1 | 15 | 50.05 | 3263.442 | 5.41349 |
| MMP2 | 14 | 53.28333 | 6415.73 | 5.65739 |
| ALOX15 | 13 | 39.67421 | 1051.667 | 4.34776 |
| IL1B | 12 | 51.3619 | 5683.476 | 5.4347 |
| MMP1 | 12 | 50.1119 | 2907.705 | 5.37637 |
| PTGS1 | 12 | 39.50754 | 103.6667 | 4.35306 |
| HSP90AB1 | 12 | 41.68333 | 131.3952 | 4.83025 |
| PTGES | 11 | 43.35476 | 1246 | 4.88858 |
| TIMP2 | 11 | 47.2619 | 746.8599 | 5.23322 |
| F2R | 11 | 41.42143 | 2546 | 4.77193 |
| NCOA2 | 11 | 46.93333 | 1075.833 | 5.11657 |
| NCOA3 | 11 | 48.21667 | 2225.083 | 5.40288 |
| MMP7 | 10 | 47.44524 | 107.7222 | 5.31275 |
| NCOR1 | 10 | 46.85 | 1483.846 | 5.12717 |
| NR1H4 | 10 | 42.01548 | 595.3963 | 4.65528 |
| SRC | 10 | 50.15 | 9047.067 | 5.61497 |
| NOS3 | 10 | 39.36667 | 268 | 4.76133 |
| MMP8 | 10 | 41.92143 | 1468.722 | 4.79844 |
| C5AR1 | 10 | 10 | 43.33333 | 0.13316 |

**Table ST3: GO analysis of top biological processes**

| **GO ID** | **GO Description** | **P-value** | **Corrected p value** | **Cluster frequency** | **Total frequency** | **Genes** |
| --- | --- | --- | --- | --- | --- | --- |
| 48518 | positive regulation of biological process | 8.17E-09 | 9.02E-06 | 16/23 69.5% | 2208/14305 15.4% | SREBF1 HSP90AA1 HSP90AB1 SRC NOS3 NCOA3 NR1H2 F2R NR1H4 PTGS2 ESR1 MMP9 PTGS1 RXRA IL1B TIMP1 |
| 10033 | response to organic substance | 2.69E-08 | 1.12E-05 | 11/23 47.8% | 869/14305 6.0% | HSP90AA1 HSP90AB1 RXRA SRC NOS3 IL1B F2R NR1H4 PTGS2 ESR1 PTGS1 |
| 51173 | positive regulation of nitrogen compound metabolic process | 3.45E-08 | 1.12E-05 | 10/23 43.4% | 677/14305 4.7% | SREBF1 HSP90AA1 HSP90AB1 RXRA NCOA3 NR1H2 IL1B F2R NR1H4 PTGS2 |
| 42221 | response to chemical stimulus | 5.53E-08 | 1.12E-05 | 13/23 56.5% | 1465/14305 10.2% | HSP90AA1 HSP90AB1 TRPA1 SRC NOS3 MMP2 F2R NR1H4 PTGS2 ESR1 PTGS1 RXRA IL1B |
| 31328 | positive regulation of cellular biosynthetic process | 6.18E-08 | 1.12E-05 | 10/23 43.4% | 720/14305 5.0% | SREBF1 HSP90AA1 HSP90AB1 RXRA NCOA3 NR1H2 IL1B F2R NR1H4 PTGS2 |
| 45429 | positive regulation of nitric oxide biosynthetic process | 6.28E-08 | 1.12E-05 | 4/23 17.3% | 25/14305 0.1% | HSP90AA1 HSP90AB1 IL1B PTGS2 |
| 9891 | positive regulation of biosynthetic process | 7.13E-08 | 1.12E-05 | 10/23 43.4% | 731/14305 5.1% | SREBF1 HSP90AA1 HSP90AB1 RXRA NCOA3 NR1H2 IL1B F2R NR1H4 PTGS2 |
| 30522 | intracellular receptor mediated signaling pathway | 1.24E-07 | 1.68E-05 | 5/23 21.7% | 77/14305 0.5% | NCOA2 RXRA NCOA3 NR1H2 ESR1 |
| 9893 | positive regulation of metabolic process | 1.37E-07 | 1.68E-05 | 11/23 47.8% | 1018/14305 7.1% | SREBF1 HSP90AA1 HSP90AB1 RXRA NCOA3 NR1H2 IL1B F2R NR1H4 PTGS2 ESR1 |
| 45428 | regulation of nitric oxide biosynthetic process | 1.55E-07 | 1.71E-05 | 4/23 17.3% | 31/14305 0.2% | HSP90AA1 HSP90AB1 IL1B PTGS2 |
| 42127 | regulation of cell proliferation | 2.87E-07 | 2.88E-05 | 10/23 43.4% | 848/14305 5.9% | RXRA NOS3 IL1B F2R TIMP2 TIMP1 PTGS2 ESR1 PTGS1 PTGES |
| 19369 | arachidonic acid metabolic process | 5.94E-07 | 5.47E-05 | 3/23 13.0% | 11/14305 0.0% | ALOX15 PTGS2 PTGS1 |
| 48523 | negative regulation of cellular process | 8.47E-07 | 7.09E-05 | 13/23 56.5% | 1844/14305 12.8% | HSP90AB1 NOS3 NR1H2 F2R NR1H4 PTGS2 ESR1 PTGS1 RXRA IL1B TIMP2 TIMP1 PTGES |
| 31325 | positive regulation of cellular metabolic process | 9.58E-07 | 7.09E-05 | 10/23 43.4% | 966/14305 6.7% | SREBF1 HSP90AA1 HSP90AB1 RXRA NCOA3 NR1H2 IL1B F2R NR1H4 PTGS2 |
| 32496 | response to lipopolysaccharide | 9.63E-07 | 7.09E-05 | 5/23 21.7% | 116/14305 0.8% | NOS3 IL1B F2R NR1H4 PTGS2 |
| 6690 | icosanoid metabolic process | 1.11E-06 | 7.42E-05 | 4/23 17.3% | 50/14305 0.3% | ALOX15 PTGS2 PTGS1 PTGES |
| 48545 | response to steroid hormone stimulus | 1.14E-06 | 7.42E-05 | 6/23 26.0% | 225/14305 1.5% | RXRA NOS3 IL1B PTGS2 ESR1 PTGS1 |
| 33559 | unsaturated fatty acid metabolic process | 1.41E-06 | 8.33E-05 | 4/23 17.3% | 53/14305 0.3% | ALOX15 PTGS2 PTGS1 PTGES |
| 8285 | negative regulation of cell proliferation | 1.47E-06 | 8.33E-05 | 7/23 30.4% | 379/14305 2.6% | RXRA NOS3 IL1B F2R TIMP2 PTGS2 PTGES |
| 2237 | response to molecule of bacterial origin | 1.51E-06 | 8.33E-05 | 5/23 21.7% | 127/14305 0.8% | NOS3 IL1B F2R NR1H4 PTGS2 |
| 51704 | multi-organism process | 1.76E-06 | 9.23E-05 | 9/23 39.1% | 785/14305 5.4% | RXRA SRC NOS3 MMP1 IL1B F2R NR1H4 PTGS2 ESR1 |
| 45907 | positive regulation of vasoconstriction | 2.01E-06 | 1.01E-04 | 3/23 13.0% | 16/14305 0.1% | F2R PTGS2 PTGS1 |
| 48522 | positive regulation of cellular process | 2.23E-06 | 1.07E-04 | 13/23 56.5% | 2004/14305 14.0% | SREBF1 HSP90AA1 HSP90AB1 NCOA3 NR1H2 F2R NR1H4 PTGS2 ESR1 MMP9 RXRA IL1B TIMP1 |
| 33280 | response to vitamin D | 2.43E-06 | 1.07E-04 | 3/23 13.0% | 17/14305 0.1% | RXRA IL1B PTGS2 |
| 48519 | negative regulation of biological process | 2.44E-06 | 1.07E-04 | 13/23 56.5% | 2020/14305 14.1% | HSP90AB1 NOS3 NR1H2 F2R NR1H4 PTGS2 ESR1 PTGS1 RXRA IL1B TIMP2 TIMP1 PTGES |
| 10628 | positive regulation of gene expression | 2.66E-06 | 1.07E-04 | 8/23 34.7% | 603/14305 4.2% | SREBF1 RXRA NCOA3 NR1H2 IL1B F2R NR1H4 ESR1 |
| 32355 | response to estradiol stimulus | 2.84E-06 | 1.07E-04 | 4/23 17.3% | 63/14305 0.4% | NOS3 IL1B PTGS2 ESR1 |
| 6629 | lipid metabolic process | 2.90E-06 | 1.07E-04 | 9/23 39.1% | 834/14305 5.8% | SREBF1 RXRA NR1H2 ALOX15 NR1H4 PTGS2 ESR1 PTGS1 PTGES |
| 30728 | ovulation | 2.92E-06 | 1.07E-04 | 3/23 13.0% | 18/14305 0.1% | NOS3 IL1B PTGS2 |
| 45987 | positive regulation of smooth muscle contraction | 2.92E-06 | 1.07E-04 | 3/23 13.0% | 18/14305 0.1% | F2R PTGS2 PTGS1 |
| 32501 | multicellular organismal process | 3.29E-06 | 1.17E-04 | 18/23 78.2% | 4375/14305 30.5% | HSP90AB1 TRPA1 SRC NOS3 MMP1 MMP2 NCOA3 ALOX15 F2R NR1H4 PTGS2 ESR1 MMP9 PTGS1 RXRA IL1B TIMP2 TIMP1 |
| 30162 | regulation of proteolysis | 3.43E-06 | 1.18E-04 | 4/23 17.3% | 66/14305 0.4% | HSP90AB1 NR1H2 IL1B TIMP1 |
| 9607 | response to biotic stimulus | 4.41E-06 | 1.41E-04 | 7/23 30.4% | 447/14305 3.1% | HSP90AA1 HSP90AB1 NOS3 IL1B F2R NR1H4 PTGS2 |
| 6950 | response to stress | 4.58E-06 | 1.41E-04 | 12/23 52.1% | 1773/14305 12.3% | SREBF1 HSP90AA1 HSP90AB1 RXRA TRPA1 NOS3 MMP2 IL1B ALOX15 F2R PTGS2 PTGS1 |
| 6692 | prostanoid metabolic process | 4.74E-06 | 1.41E-04 | 3/23 13.0% | 21/14305 0.1% | PTGS2 PTGS1 PTGES |
| 6693 | prostaglandin metabolic process | 4.74E-06 | 1.41E-04 | 3/23 13.0% | 21/14305 0.1% | PTGS2 PTGS1 PTGES |
| 30574 | collagen catabolic process | 4.74E-06 | 1.41E-04 | 3/23 13.0% | 21/14305 0.1% | MMP1 MMP2 MMP9 |
| 51051 | negative regulation of transport | 4.85E-06 | 1.41E-04 | 5/23 21.7% | 161/14305 1.1% | NOS3 NR1H2 IL1B PTGS2 PTGS1 |
| 45933 | positive regulation of muscle contraction | 6.30E-06 | 1.75E-04 | 3/23 13.0% | 23/14305 0.1% | F2R PTGS2 PTGS1 |
| 10243 | response to organic nitrogen | 6.36E-06 | 1.75E-04 | 4/23 17.3% | 77/14305 0.5% | NOS3 IL1B PTGS2 PTGS1 |
| 45861 | negative regulation of proteolysis | 7.19E-06 | 1.94E-04 | 3/23 13.0% | 24/14305 0.1% | HSP90AB1 NR1H2 TIMP1 |
| 19371 | cyclooxygenase pathway | 7.41E-06 | 1.95E-04 | 2/23 8.6% | 3/14305 0.0% | PTGS2 PTGS1 |
| 38 | very long-chain fatty acid metabolic process | 8.16E-06 | 2.10E-04 | 3/23 13.0% | 25/14305 0.1% | ALOX15 PTGS2 PTGS1 |
| 90257 | regulation of muscle system process | 9.00E-06 | 2.26E-04 | 4/23 17.3% | 84/14305 0.5% | NOS3 F2R PTGS2 PTGS1 |
| 45893 | positive regulation of transcription, DNA-dependent | 9.20E-06 | 2.26E-04 | 7/23 30.4% | 500/14305 3.4% | SREBF1 RXRA NCOA3 NR1H2 IL1B F2R NR1H4 |
| 51254 | positive regulation of RNA metabolic process | 9.95E-06 | 2.39E-04 | 7/23 30.4% | 506/14305 3.5% | SREBF1 RXRA NCOA3 NR1H2 IL1B F2R NR1H4 |
| 44243 | multicellular organismal catabolic process | 1.04E-05 | 2.43E-04 | 3/23 13.0% | 27/14305 0.1% | MMP1 MMP2 MMP9 |
| 32963 | collagen metabolic process | 1.43E-05 | 3.22E-04 | 3/23 13.0% | 30/14305 0.2% | MMP1 MMP2 MMP9 |
| 14805 | smooth muscle adaptation | 1.48E-05 | 3.22E-04 | 2/23 8.6% | 4/14305 0.0% | NOS3 IL1B |
| 60068 | vagina development | 1.48E-05 | 3.22E-04 | 2/23 8.6% | 4/14305 0.0% | NCOA3 ESR1 |
| 32502 | developmental process | 1.49E-05 | 3.22E-04 | 15/23 65.2% | 3234/14305 22.6% | HSP90AB1 NOS3 MMP2 NCOA3 ALOX15 F2R NR1H4 PTGS2 ESR1 MMP9 PTGS1 RXRA IL1B TIMP2 TIMP1 |
| 51384 | response to glucocorticoid stimulus | 1.59E-05 | 3.38E-04 | 4/23 17.3% | 97/14305 0.6% | RXRA IL1B PTGS2 PTGS1 |
| 44259 | multicellular organismal macromolecule metabolic process | 1.92E-05 | 3.86E-04 | 3/23 13.0% | 33/14305 0.2% | MMP1 MMP2 MMP9 |
| 46456 | icosanoid biosynthetic process | 1.92E-05 | 3.86E-04 | 3/23 13.0% | 33/14305 0.2% | ALOX15 PTGS2 PTGS1 |
| 48731 | system development | 1.92E-05 | 3.86E-04 | 13/23 56.5% | 2422/14305 16.9% | HSP90AB1 NOS3 MMP2 NCOA3 ALOX15 F2R NR1H4 PTGS2 ESR1 MMP9 RXRA TIMP2 TIMP1 |
| 31960 | response to corticosteroid stimulus | 2.10E-05 | 4.14E-04 | 4/23 17.3% | 104/14305 0.7% | RXRA IL1B PTGS2 PTGS1 |
| 51239 | regulation of multicellular organismal process | 2.17E-05 | 4.20E-04 | 9/23 39.1% | 1067/14305 7.4% | RXRA SRC NOS3 IL1B F2R TIMP2 PTGS2 ESR1 PTGS1 |
| 45941 | positive regulation of transcription | 2.28E-05 | 4.35E-04 | 7/23 30.4% | 575/14305 4.0% | SREBF1 RXRA NCOA3 NR1H2 IL1B F2R NR1H4 |
| 6636 | unsaturated fatty acid biosynthetic process | 2.50E-05 | 4.61E-04 | 3/23 13.0% | 36/14305 0.2% | ALOX15 PTGS2 PTGS1 |
| 19229 | regulation of vasoconstriction | 2.50E-05 | 4.61E-04 | 3/23 13.0% | 36/14305 0.2% | F2R PTGS2 PTGS1 |
| 45944 | positive regulation of transcription from RNA polymerase II promoter | 2.61E-05 | 4.73E-04 | 6/23 26.0% | 388/14305 2.7% | SREBF1 RXRA NCOA3 NR1H2 IL1B NR1H4 |
| 9617 | response to bacterium | 2.79E-05 | 4.97E-04 | 5/23 21.7% | 231/14305 1.6% | NOS3 IL1B F2R NR1H4 PTGS2 |
| 6940 | regulation of smooth muscle contraction | 2.95E-05 | 5.17E-04 | 3/23 13.0% | 38/14305 0.2% | F2R PTGS2 PTGS1 |
| 43627 | response to estrogen stimulus | 3.01E-05 | 5.20E-04 | 4/23 17.3% | 114/14305 0.7% | NOS3 IL1B PTGS2 ESR1 |
| 44236 | multicellular organismal metabolic process | 3.20E-05 | 5.34E-04 | 3/23 13.0% | 39/14305 0.2% | MMP1 MMP2 MMP9 |
| 1503 | ossification | 3.23E-05 | 5.34E-04 | 4/23 17.3% | 116/14305 0.8% | MMP2 ALOX15 PTGS2 ESR1 |
| 7275 | multicellular organismal development | 3.24E-05 | 5.34E-04 | 14/23 60.8% | 2971/14305 20.7% | HSP90AB1 NOS3 MMP2 NCOA3 ALOX15 F2R NR1H4 PTGS2 ESR1 MMP9 RXRA IL1B TIMP2 TIMP1 |
| 48513 | organ development | 3.69E-05 | 5.91E-04 | 11/23 47.8% | 1792/14305 12.5% | HSP90AB1 RXRA NOS3 MMP2 NCOA3 ALOX15 F2R TIMP1 PTGS2 ESR1 MMP9 |
| 60687 | regulation of branching involved in prostate gland morphogenesis | 3.69E-05 | 5.91E-04 | 2/23 8.6% | 6/14305 0.0% | RXRA ESR1 |
| 7565 | female pregnancy | 3.93E-05 | 6.20E-04 | 4/23 17.3% | 122/14305 0.8% | RXRA IL1B PTGS2 ESR1 |
| 51171 | regulation of nitrogen compound metabolic process | 4.26E-05 | 6.59E-04 | 14/23 60.8% | 3040/14305 21.2% | SREBF1 NCOA2 HSP90AA1 HSP90AB1 NOS3 NCOA3 NR1H2 F2R NR1H4 PTGS2 ESR1 RXRA IL1B TIMP2 |
| 45766 | positive regulation of angiogenesis | 4.30E-05 | 6.59E-04 | 3/23 13.0% | 43/14305 0.3% | NOS3 IL1B PTGS2 |
| 60348 | bone development | 5.20E-05 | 7.79E-04 | 4/23 17.3% | 131/14305 0.9% | MMP2 ALOX15 PTGS2 ESR1 |
| 45935 | positive regulation of nucleobase, nucleoside, nucleotide and nucleic acid metabolic process | 5.32E-05 | 7.79E-04 | 7/23 30.4% | 656/14305 4.5% | SREBF1 RXRA NCOA3 NR1H2 IL1B F2R NR1H4 |
| 48856 | anatomical structure development | 5.34E-05 | 7.79E-04 | 13/23 56.5% | 2656/14305 18.5% | HSP90AB1 NOS3 MMP2 NCOA3 ALOX15 F2R NR1H4 PTGS2 ESR1 MMP9 RXRA TIMP2 TIMP1 |
| 9725 | response to hormone stimulus | 5.36E-05 | 7.79E-04 | 6/23 26.0% | 441/14305 3.0% | RXRA NOS3 IL1B PTGS2 ESR1 PTGS1 |
| 32844 | regulation of homeostatic process | 5.51E-05 | 7.91E-04 | 4/23 17.3% | 133/14305 0.9% | SRC IL1B F2R PTGS2 |
| 9895 | negative regulation of catabolic process | 5.62E-05 | 7.96E-04 | 3/23 13.0% | 47/14305 0.3% | HSP90AB1 IL1B TIMP1 |
| 32846 | positive regulation of homeostatic process | 6.37E-05 | 8.79E-04 | 3/23 13.0% | 49/14305 0.3% | IL1B F2R PTGS2 |
| 10557 | positive regulation of macromolecule biosynthetic process | 6.38E-05 | 8.79E-04 | 7/23 30.4% | 675/14305 4.7% | SREBF1 RXRA NCOA3 NR1H2 IL1B F2R NR1H4 |
| 44238 | primary metabolic process | 6.45E-05 | 8.79E-04 | 18/23 78.2% | 5288/14305 36.9% | SREBF1 HSP90AA1 HSP90AB1 SRC NOS3 MMP1 MMP2 NR1H2 ALOX15 F2R NR1H4 PTGS2 ESR1 MMP9 PTGS1 RXRA IL1B PTGES |
| 50896 | response to stimulus | 6.55E-05 | 8.81E-04 | 15/23 65.2% | 3633/14305 25.3% | SREBF1 HSP90AA1 HSP90AB1 TRPA1 SRC NOS3 MMP2 ALOX15 F2R NR1H4 PTGS2 ESR1 PTGS1 RXRA IL1B |
| 51240 | positive regulation of multicellular organismal process | 6.75E-05 | 8.98E-04 | 5/23 21.7% | 278/14305 1.9% | NOS3 IL1B F2R PTGS2 PTGS1 |
| 10604 | positive regulation of macromolecule metabolic process | 6.85E-05 | 9.01E-04 | 8/23 34.7% | 941/14305 6.5% | SREBF1 RXRA NCOA3 NR1H2 IL1B F2R NR1H4 ESR1 |
| 45834 | positive regulation of lipid metabolic process | 7.19E-05 | 9.34E-04 | 3/23 13.0% | 51/14305 0.3% | NR1H2 IL1B PTGS2 |
| 7568 | aging | 7.72E-05 | 9.91E-04 | 4/23 17.3% | 145/14305 1.0% | RXRA NOS3 IL1B PTGS1 |

**Table ST4: GO analysis of top cellular components**

| **GO ID** | **GO Description** | **P-value** | **Corrected p value** | **Cluster frequency** | **Total frequency** | **Genes** |
| --- | --- | --- | --- | --- | --- | --- |
| 5901 | caveola | 6.61E-07 | 7.14E-05 | 4/24 16.6% | 48/16378 0.2% | SRC NOS3 F2R PTGS2 |
| 5578 | proteinaceous extracellular matrix | 4.59E-06 | 2.48E-04 | 6/24 25.0% | 312/16378 1.9% | MMP1 MMP2 TIMP2 TIMP1 MMP8 MMP9 |
| 31012 | extracellular matrix | 7.52E-06 | 2.71E-04 | 6/24 25.0% | 340/16378 2.0% | MMP1 MMP2 TIMP2 TIMP1 MMP8 MMP9 |
| 45121 | membrane raft | 5.91E-05 | 1.60E-03 | 4/24 16.6% | 148/16378 0.9% | SRC NOS3 F2R PTGS2 |
| 44421 | extracellular region part | 3.90E-04 | 8.43E-03 | 7/24 29.1% | 985/16378 6.0% | MMP1 MMP2 IL1B TIMP2 TIMP1 MMP8 MMP9 |

**Table ST5: GO analysis of top molecular functions**

| **GO ID** | **GO Description** | **p-value** | **Corrected p value** | **Cluster frequency** | **Total frequency** | **Genes** |
| --- | --- | --- | --- | --- | --- | --- |
| 30235 | nitric-oxide synthase regulator activity | 3.29E-08 | 5.86E-06 | 3/24 12.5% | 5/15443 0.0% | HSP90AA1 HSP90AB1 ESR1 |
| 35257 | nuclear hormone receptor binding | 1.21E-07 | 1.08E-05 | 5/24 20.8% | 79/15443 0.5% | NCOA2 RXRA NCOA3 NR1H2 NR1H4 |
| 51427 | hormone receptor binding | 2.34E-07 | 1.39E-05 | 5/24 20.8% | 90/15443 0.5% | NCOA2 RXRA NCOA3 NR1H2 NR1H4 |
| 3707 | steroid hormone receptor activity | 9.07E-07 | 4.04E-05 | 4/24 16.6% | 49/15443 0.3% | RXRA NR1H2 NR1H4 ESR1 |
| 4879 | ligand-dependent nuclear receptor activity | 1.16E-06 | 4.11E-05 | 4/24 16.6% | 52/15443 0.3% | RXRA NR1H2 NR1H4 ESR1 |
| 4666 | prostaglandin-endoperoxide synthase activity | 2.31E-06 | 6.87E-05 | 2/24 8.3% | 2/15443 0.0% | PTGS2 PTGS1 |
| 5102 | receptor binding | 5.36E-06 | 1.36E-04 | 9/24 37.5% | 922/15443 5.9% | NCOA2 RXRA SRC NCOA3 NR1H2 IL1B F2R TIMP2 NR1H4 |
| 30911 | TPR domain binding | 6.94E-06 | 1.54E-04 | 2/24 8.3% | 3/15443 0.0% | HSP90AA1 HSP90AB1 |
| 4222 | metalloendopeptidase activity | 1.79E-05 | 3.54E-04 | 4/24 16.6% | 103/15443 0.6% | MMP1 MMP2 MMP8 MMP9 |
| 46914 | transition metal ion binding | 4.97E-05 | 8.84E-04 | 12/24 50.0% | 2280/15443 14.7% | RXRA MMP1 NOS3 MMP2 NR1H2 ALOX15 NR1H4 PTGS2 MMP8 MMP9 ESR1 PTGS1 |
| 46965 | retinoid X receptor binding | 8.28E-05 | 1.31E-03 | 2/24 8.3% | 9/15443 0.0% | NR1H2 NR1H4 |
| 10576 | metalloenzyme regulator activity | 1.03E-04 | 1.31E-03 | 2/24 8.3% | 10/15443 0.0% | TIMP2 TIMP1 |
| 48551 | metalloenzyme inhibitor activity | 1.03E-04 | 1.31E-03 | 2/24 8.3% | 10/15443 0.0% | TIMP2 TIMP1 |
| 8191 | metalloendopeptidase inhibitor activity | 1.03E-04 | 1.31E-03 | 2/24 8.3% | 10/15443 0.0% | TIMP2 TIMP1 |
| 8134 | transcription factor binding | 1.16E-04 | 1.38E-03 | 6/24 25.0% | 522/15443 3.3% | NCOA2 RXRA NCOA3 NR1H2 NR1H4 ESR1 |
| 42974 | retinoic acid receptor binding | 1.26E-04 | 1.40E-03 | 2/24 8.3% | 11/15443 0.0% | NR1H2 NR1H4 |
| 5496 | steroid binding | 1.42E-04 | 1.48E-03 | 3/24 12.5% | 66/15443 0.4% | RXRA NR1H4 ESR1 |
| 16922 | ligand-dependent nuclear receptor binding | 1.51E-04 | 1.50E-03 | 2/24 8.3% | 12/15443 0.0% | NCOA2 NCOA3 |
| 8237 | metallopeptidase activity | 1.65E-04 | 1.55E-03 | 4/24 16.6% | 182/15443 1.1% | MMP1 MMP2 MMP8 MMP9 |
| 51213 | dioxygenase activity | 2.24E-04 | 1.88E-03 | 3/24 12.5% | 77/15443 0.4% | ALOX15 PTGS2 PTGS1 |
| 16702 | oxidoreductase activity, acting on single donors with incorporation of molecular oxygen, incorporation of two atoms of oxygen | 2.24E-04 | 1.88E-03 | 3/24 12.5% | 77/15443 0.4% | ALOX15 PTGS2 PTGS1 |
| 16701 | oxidoreductase activity, acting on single donors with incorporation of molecular oxygen | 2.32E-04 | 1.88E-03 | 3/24 12.5% | 78/15443 0.5% | ALOX15 PTGS2 PTGS1 |
| 5506 | iron ion binding | 2.65E-04 | 2.05E-03 | 4/24 16.6% | 206/15443 1.3% | NOS3 ALOX15 PTGS2 PTGS1 |
| 3713 | transcription coactivator activity | 3.12E-04 | 2.31E-03 | 4/24 16.6% | 215/15443 1.3% | NCOA2 RXRA NCOA3 NR1H4 |
| 8289 | lipid binding | 3.65E-04 | 2.60E-03 | 5/24 20.8% | 411/15443 2.6% | RXRA NR1H4 PTGS2 ESR1 PTGS1 |
| 46872 | metal ion binding | 4.07E-04 | 2.79E-03 | 14/24 58.3% | 3778/15443 24.4% | MMP1 NOS3 MMP2 NR1H2 ALOX15 NR1H4 PTGS2 MMP8 MMP9 ESR1 PTGS1 RXRA TIMP2 TIMP1 |
| 16563 | transcription activator activity | 4.44E-04 | 2.88E-03 | 5/24 20.8% | 429/15443 2.7% | NCOA2 RXRA NCOA3 NR1H2 NR1H4 |
| 43169 | cation binding | 4.56E-04 | 2.88E-03 | 14/24 58.3% | 3817/15443 24.7% | MMP1 NOS3 MMP2 NR1H2 ALOX15 NR1H4 PTGS2 MMP8 MMP9 ESR1 PTGS1 RXRA TIMP2 TIMP1 |
| 43167 | ion binding | 4.70E-04 | 2.88E-03 | 14/24 58.3% | 3827/15443 24.7% | MMP1 NOS3 MMP2 NR1H2 ALOX15 NR1H4 PTGS2 MMP8 MMP9 ESR1 PTGS1 RXRA TIMP2 TIMP1 |
| 20037 | heme binding | 8.62E-04 | 5.12E-03 | 3/24 12.5% | 122/15443 0.7% | NOS3 PTGS2 PTGS1 |
| 46906 | tetrapyrrole binding | 1.04E-03 | 5.65E-03 | 3/24 12.5% | 130/15443 0.8% | NOS3 PTGS2 PTGS1 |
| 16684 | oxidoreductase activity, acting on peroxide as acceptor | 1.05E-03 | 5.65E-03 | 2/24 8.3% | 31/15443 0.2% | PTGS2 PTGS1 |
| 4601 | peroxidase activity | 1.05E-03 | 5.65E-03 | 2/24 8.3% | 31/15443 0.2% | PTGS2 PTGS1 |
| 16705 | oxidoreductase activity, acting on paired donors, with incorporation or reduction of molecular oxygen | 1.18E-03 | 6.18E-03 | 3/24 12.5% | 136/15443 0.8% | NOS3 PTGS2 PTGS1 |
| 34056 | estrogen response element binding | 1.55E-03 | 7.90E-03 | 1/24 4.1% | 1/15443 0.0% | ESR1 |
| 8270 | zinc ion binding | 2.16E-03 | 1.06E-02 | 9/24 37.5% | 2007/15443 12.9% | RXRA MMP1 NOS3 MMP2 NR1H2 NR1H4 MMP8 MMP9 ESR1 |
| 3712 | transcription cofactor activity | 2.20E-03 | 1.06E-02 | 4/24 16.6% | 363/15443 2.3% | NCOA2 RXRA NCOA3 NR1H4 |
| 16209 | antioxidant activity | 2.40E-03 | 1.11E-02 | 2/24 8.3% | 47/15443 0.3% | PTGS2 PTGS1 |
| 4175 | endopeptidase activity | 2.43E-03 | 1.11E-02 | 4/24 16.6% | 373/15443 2.4% | MMP1 MMP2 MMP8 MMP9 |
| 19904 | protein domain specific binding | 2.80E-03 | 1.25E-02 | 4/24 16.6% | 388/15443 2.5% | HSP90AA1 HSP90AB1 SRC IL1B |
| 50473 | arachidonate 15-lipoxygenase activity | 3.11E-03 | 1.26E-02 | 1/24 4.1% | 2/15443 0.0% | ALOX15 |
| 32810 | sterol response element binding | 3.11E-03 | 1.26E-02 | 1/24 4.1% | 2/15443 0.0% | SREBF1 |
| 30284 | estrogen receptor activity | 3.11E-03 | 1.26E-02 | 1/24 4.1% | 2/15443 0.0% | ESR1 |
| 47977 | hepoxilin-epoxide hydrolase activity | 3.11E-03 | 1.26E-02 | 1/24 4.1% | 2/15443 0.0% | ALOX15 |
| 5178 | integrin binding | 4.27E-03 | 1.66E-02 | 2/24 8.3% | 63/15443 0.4% | SRC TIMP2 |
| 4886 | retinoid-X receptor activity | 4.66E-03 | 1.66E-02 | 1/24 4.1% | 3/15443 0.0% | RXRA |
| 50220 | prostaglandin-E synthase activity | 4.66E-03 | 1.66E-02 | 1/24 4.1% | 3/15443 0.0% | PTGES |
| 34617 | tetrahydrobiopterin binding | 4.66E-03 | 1.66E-02 | 1/24 4.1% | 3/15443 0.0% | NOS3 |
| 4517 | nitric-oxide synthase activity | 4.66E-03 | 1.66E-02 | 1/24 4.1% | 3/15443 0.0% | NOS3 |
| 4052 | arachidonate 12-lipoxygenase activity | 4.66E-03 | 1.66E-02 | 1/24 4.1% | 3/15443 0.0% | ALOX15 |
| 32403 | protein complex binding | 5.76E-03 | 2.01E-02 | 3/24 12.5% | 238/15443 1.5% | SRC TIMP2 ESR1 |
| 34618 | arginine binding | 6.20E-03 | 2.12E-02 | 1/24 4.1% | 4/15443 0.0% | NOS3 |
| 30528 | transcription regulator activity | 6.48E-03 | 2.18E-02 | 7/24 29.1% | 1507/15443 9.7% | SREBF1 NCOA2 RXRA NCOA3 NR1H2 NR1H4 ESR1 |
| 15057 | thrombin receptor activity | 7.75E-03 | 2.55E-02 | 1/24 4.1% | 5/15443 0.0% | F2R |
| 30234 | enzyme regulator activity | 9.28E-03 | 2.80E-02 | 5/24 20.8% | 860/15443 5.5% | HSP90AA1 HSP90AB1 TIMP2 TIMP1 ESR1 |
| 16165 | lipoxygenase activity | 9.29E-03 | 2.80E-02 | 1/24 4.1% | 6/15443 0.0% | ALOX15 |
| 32052 | bile acid binding | 9.29E-03 | 2.80E-02 | 1/24 4.1% | 6/15443 0.0% | NR1H4 |
| 3708 | retinoic acid receptor activity | 9.29E-03 | 2.80E-02 | 1/24 4.1% | 6/15443 0.0% | RXRA |
| 16803 | ether hydrolase activity | 9.29E-03 | 2.80E-02 | 1/24 4.1% | 6/15443 0.0% | ALOX15 |
| 3690 | double-stranded DNA binding | 9.46E-03 | 2.81E-02 | 2/24 8.3% | 95/15443 0.6% | RXRA NR1H4 |
| 70011 | peptidase activity, acting on L-amino acid peptides | 9.65E-03 | 2.82E-02 | 4/24 16.6% | 551/15443 3.5% | MMP1 MMP2 MMP8 MMP9 |
| 46870 | cadmium ion binding | 1.08E-02 | 3.06E-02 | 1/24 4.1% | 7/15443 0.0% | NOS3 |
| 8233 | peptidase activity | 1.08E-02 | 3.06E-02 | 4/24 16.6% | 570/15443 3.6% | MMP1 MMP2 MMP8 MMP9 |
| 4871 | signal transducer activity | 1.21E-02 | 3.33E-02 | 8/24 33.3% | 2128/15443 13.7% | NCOA2 RXRA SRC NCOA3 NR1H2 F2R NR1H4 ESR1 |
| 60089 | molecular transducer activity | 1.21E-02 | 3.33E-02 | 8/24 33.3% | 2128/15443 13.7% | NCOA2 RXRA SRC NCOA3 NR1H2 F2R NR1H4 ESR1 |
| 3785 | actin monomer binding | 1.24E-02 | 3.34E-02 | 1/24 4.1% | 8/15443 0.0% | NOS3 |
| 10843 | promoter binding | 1.32E-02 | 3.45E-02 | 2/24 8.3% | 113/15443 0.7% | SREBF1 ESR1 |
| 51082 | unfolded protein binding | 1.32E-02 | 3.45E-02 | 2/24 8.3% | 113/15443 0.7% | HSP90AA1 HSP90AB1 |
| 3706 | ligand-regulated transcription factor activity | 1.39E-02 | 3.48E-02 | 1/24 4.1% | 9/15443 0.0% | RXRA |
| 16801 | hydrolase activity, acting on ether bonds | 1.39E-02 | 3.48E-02 | 1/24 4.1% | 9/15443 0.0% | ALOX15 |
| 3700 | transcription factor activity | 1.39E-02 | 3.48E-02 | 5/24 20.8% | 950/15443 6.1% | SREBF1 RXRA NR1H2 NR1H4 ESR1 |
| 44212 | DNA regulatory region binding | 1.41E-02 | 3.48E-02 | 2/24 8.3% | 117/15443 0.7% | SREBF1 ESR1 |
| 43565 | sequence-specific DNA binding | 1.55E-02 | 3.77E-02 | 4/24 16.6% | 633/15443 4.0% | SREBF1 NR1H2 NR1H4 ESR1 |
| 5149 | interleukin-1 receptor binding | 1.70E-02 | 4.08E-02 | 1/24 4.1% | 11/15443 0.0% | IL1B |
| 10181 | FMN binding | 1.85E-02 | 4.39E-02 | 1/24 4.1% | 12/15443 0.0% | NOS3 |
| 16491 | oxidoreductase activity | 2.02E-02 | 4.73E-02 | 4/24 16.6% | 686/15443 4.4% | NOS3 ALOX15 PTGS2 PTGS1 |
| 43566 | structure-specific DNA binding | 2.08E-02 | 4.82E-02 | 2/24 8.3% | 144/15443 0.9% | RXRA NR1H4 |
| 5515 | protein binding | 2.13E-02 | 4.82E-02 | 18/24 75.0% | 8123/15443 52.5% | SREBF1 NCOA2 HSP90AA1 HSP90AB1 SRC NOS3 MMP2 NCOA3 NR1H2 F2R NR1H4 PTGS2 MMP9 ESR1 RXRA IL1B TIMP2 TIMP1 |
| 4866 | endopeptidase inhibitor activity | 2.14E-02 | 4.82E-02 | 2/24 8.3% | 146/15443 0.9% | TIMP2 TIMP1 |
| 61135 | endopeptidase regulator activity | 2.17E-02 | 4.82E-02 | 2/24 8.3% | 147/15443 0.9% | TIMP2 TIMP1 |
| 31406 | carboxylic acid binding | 2.19E-02 | 4.82E-02 | 2/24 8.3% | 148/15443 0.9% | NOS3 NR1H4 |

**Table ST6: Top signaling pathways**

| **Pathway** | **ID** | **P-Value** | **Corrected P-Value** | **Input** |
| --- | --- | --- | --- | --- |
| Arachidonic acid metabolism | hsa00590 | 9.53E-08 | 1.19E-06 | PTGS2\|PTGS1\|PTGES\|ALOX15 |
| Proteoglycans in cancer | hsa05205 | 8.70E-06 | 6.49E-05 | SRC\|ESR1\|MMP2\|MMP9 |
| VEGF signaling pathway | hsa04370 | 8.41E-06 | 6.49E-05 | PTGS2\|NOS3\|SRC |
| Th17 cell differentiation | hsa04659 | 7.31E-07 | 8.18E-06 | HSP90AB1\|HSP90AA1\|IL1B\|RXRA |
| Serotonergic synapse | hsa04726 | 5.80E-05 | 0.000295 | PTGS2\|PTGS1\|ALOX15 |
| Endocrine resistance | hsa01522 | 5.73E-09 | 1.07E-07 | NCOA3\|ESR1\|MMP2\|SRC\|MMP9 |
| TNF signaling pathway | hsa04668 | 5.37E-05 | 0.000286 | PTGS2\|IL1B\|MMP9 |
| Insulin resistance | hsa04931 | 4.83E-05 | 0.000271 | SREBF1\|NOS3\|NR1H2 |
| C-type lectin receptor signaling pathway | hsa04625 | 4.33E-05 | 0.000255 | PTGS2\|IL1B\|SRC |
| Inflammatory mediator regulation of TRP channels | hsa04750 | 3.86E-05 | 0.00024 | TRPA1\|IL1B\|SRC |
| AGE-RAGE signaling pathway in diabetic complications | hsa04933 | 3.86E-05 | 0.00024 | IL1B\|NOS3\|MMP2 |
| Fluid shear stress and atherosclerosis | hsa05418 | 3.86E-12 | 1.44E-10 | SRC\|NOS3\|HSP90AB1\|MMP9\|HSP90AA1\|IL1B\|MMP2 |
| IL-17 signaling pathway | hsa04657 | 3.75E-11 | 1.05E-09 | PTGS2\|HSP90AB1\|MMP9\|HSP90AA1\|IL1B\|MMP1 |
| Necroptosis | hsa04217 | 3.63E-06 | 3.13E-05 | HSP90AB1\|HSP90AA1\|IL1B\|ALOX15 |
| Prostate cancer | hsa05215 | 3.54E-05 | 0.00024 | HSP90AB1\|HSP90AA1\|MMP9 |
| PI3K-Akt signaling pathway | hsa04151 | 2.84E-06 | 2.65E-05 | HSP90AB1\|HSP90AA1\|NOS3\|RXRA\|F2R |
| Relaxin signaling pathway | hsa04926 | 2.24E-08 | 3.14E-07 | SRC\|MMP1\|MMP2\|NOS3\|MMP9 |
| Bladder cancer | hsa05219 | 1.87E-08 | 2.99E-07 | SRC\|MMP1\|MMP2\|MMP9 |
| Thyroid hormone signaling pathway | hsa04919 | 1.55E-10 | 3.48E-09 | NCOA2\|NCOA3\|NCOA1\|SRC\|ESR1\|RXRA |
| Estrogen signaling pathway | hsa04915 | 1.32E-18 | 1.48E-16 | NCOA2\|NCOA3\|NCOA1\|ESR1\|SRC\|HSP90AB1\|NOS3\|MMP9\|HSP90AA1\|MMP2 |
| Platelet activation | hsa04611 | 1.29E-06 | 1.32E-05 | SRC\|PTGS1\|NOS3\|F2R |
| Pathways in cancer | hsa05200 | 1.15E-14 | 6.43E-13 | NCOA3\|NCOA1\|ESR1\|RXRA\|HSP90AB1\|F2R\|PTGS2\|MMP9\|HSP90AA1\|MMP2\|MMP1 |
| Yersinia infection | hsa05135 | 0.002785 | 0.007426 | SRC\|IL1B |
| Leukocyte transendothelial migration | hsa04670 | 0.002399 | 0.006554 | MMP2\|MMP9 |
| HIF-1 signaling pathway | hsa04066 | 0.002277 | 0.006375 | NOS3\|TIMP1 |
| NF-kappa B signaling pathway | hsa04064 | 0.001928 | 0.005536 | PTGS2\|IL1B |
| Progesterone-mediated oocyte maturation | hsa04914 | 0.001891 | 0.005536 | HSP90AB1\|HSP90AA1 |
| Metabolic pathways | hsa01100 | 0.001884 | 0.005536 | PTGS2\|PTGS1\|NOS3\|PTGES\|ALOX15 |
| Small cell lung cancer | hsa05222 | 0.001675 | 0.005213 | PTGS2\|RXRA |
| GnRH signaling pathway | hsa04912 | 0.001675 | 0.005213 | SRC\|MMP2 |
| Rheumatoid arthritis | hsa05323 | 0.001607 | 0.005213 | IL1B\|MMP1 |
| Antigen processing and presentation | hsa04612 | 0.001163 | 0.003948 | HSP90AB1\|HSP90AA1 |
| PPAR signaling pathway | hsa03320 | 0.001134 | 0.003948 | RXRA\|MMP1 |
| Leishmaniasis | hsa05140 | 0.001077 | 0.003892 | PTGS2\|IL1B |
| Bile secretion | hsa04976 | 0.001022 | 0.003815 | NR1H4\|RXRA |
| Prolactin signaling pathway | hsa04917 | 0.000968 | 0.003738 | SRC\|ESR1 |
| Regulation of lipolysis in adipocytes | hsa04923 | 0.000608 | 0.002431 | PTGS2\|PTGS1 |
| Human cytomegalovirus infection | hsa05163 | 0.000404 | 0.001677 | PTGS2\|IL1B\|SRC |
| NOD-like receptor signaling pathway | hsa04621 | 0.000206 | 0.000886 | HSP90AB1\|HSP90AA1\|IL1B |
| Oxytocin signaling pathway | hsa04921 | 0.000133 | 0.000594 | PTGS2\|NOS3\|SRC |
| Non-alcoholic fatty liver disease (NAFLD) | hsa04932 | 0.000123 | 0.000573 | IL1B\|SREBF1\|RXRA |
| Breast cancer | hsa05224 | 0.000118 | 0.000573 | NCOA3\|NCOA1\|ESR1 |

**Table ST7: Molecular docking results of all the 7 compounds**

| **Protein** | **Ligand** | **CDOCKER energy (kcal/mol)** | **h-bonded interactions** | **Other non-bonded interactions** |
| --- | --- | --- | --- | --- |
| **Proto-oncogene tyrosine-protein kinase Src** | 7-t-Butyl-3,3-dimethyl-1-indanone | -9.44272 | 0 | 6 |
|  | Ageratochromene | 1.67667 | 2 | 4 |
|  | Dibunol | 6.21701 | 0 | 7 |
|  | Hexa-hydro-farnesol | 17.1205 | 2 | 8 |
|  | 3,3,4,5,5,8-hexamethyl-6,7-dihydro-2*H*-s-indacen-1-one | -20.8252 | 1 | 6 |
|  | trans-Nerolidyl formate | -32.9192 | 1 | 6 |
|  | Widdrol | -31.452 | 2 | 3 |
| **Prostaglandin G/H synthase 2** | 7-t-Butyl-3,3-dimethyl-1-indanone | 0.0947657 | 1 | 13 |
|  | Ageratochromene | 5.1369 | 3 | 12 |
|  | Dibunol | 15.7634 | 1 | 14 |
|  | Hexa-hydro-farnesol | 25.094 | 1 | 18 |
|  | 3,3,4,5,5,8-hexamethyl-6,7-dihydro-2*H*-s-indacen-1-one | -14.8853 | 2 | 11 |
|  | trans-Nerolidyl formate | -24.1517 | 4 | 15 |
|  | Widdrol | -28.2505 | 2 | 9 |
| **Interleukin-1 beta** | 7-t-Butyl-3,3-dimethyl-1-indanone | -8.2164 | 0 | 9 |
|  | Ageratochromene | -3.12782 | 2 | 6 |
|  | Dibunol | 14.1995 | 1 | 7 |
|  | Hexa-hydro-farnesol | 14.0183 | 1 | 9 |
|  | 3,3,4,5,5,8-hexamethyl-6,7-dihydro-2*H*-s-indacen-1-one | -21.672 | 2 | 7 |
|  | trans-Nerolidyl formate | -32.8594 | 2 | 6 |
|  | Widdrol | -29.5415 | 1 | 7 |

**Supplementary Figures**


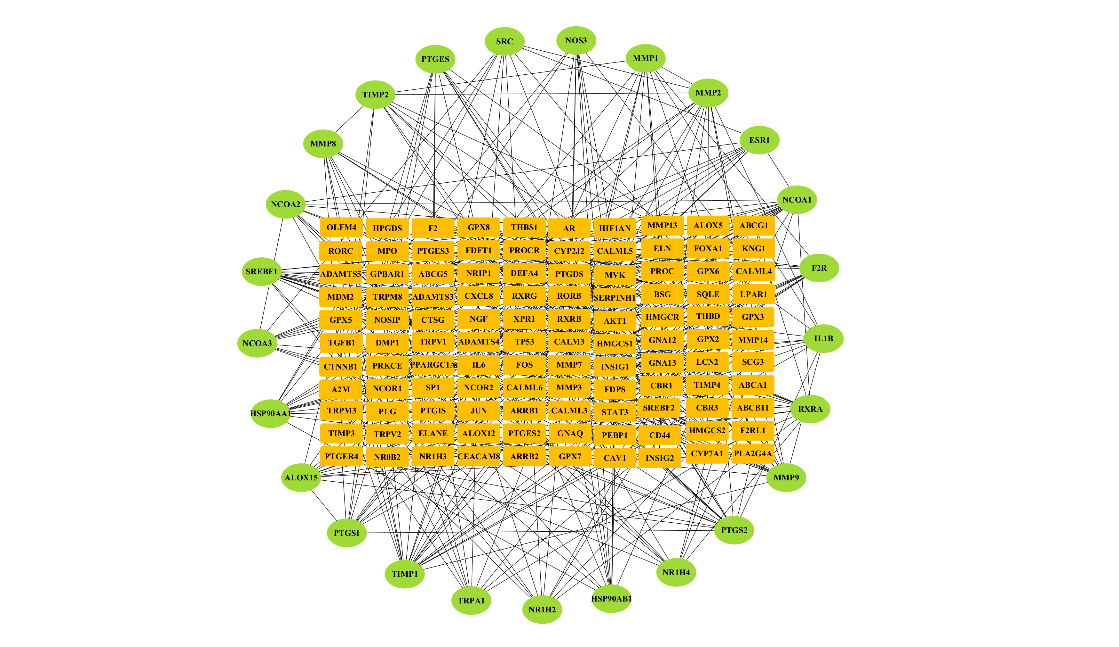


**Fig. SF1** The core targets and non-core targets network. The yellow rectangular nodes represent the 25 core targets and the green eclipses indicate the non-core targets, the node size is proportional to the target degree in the network


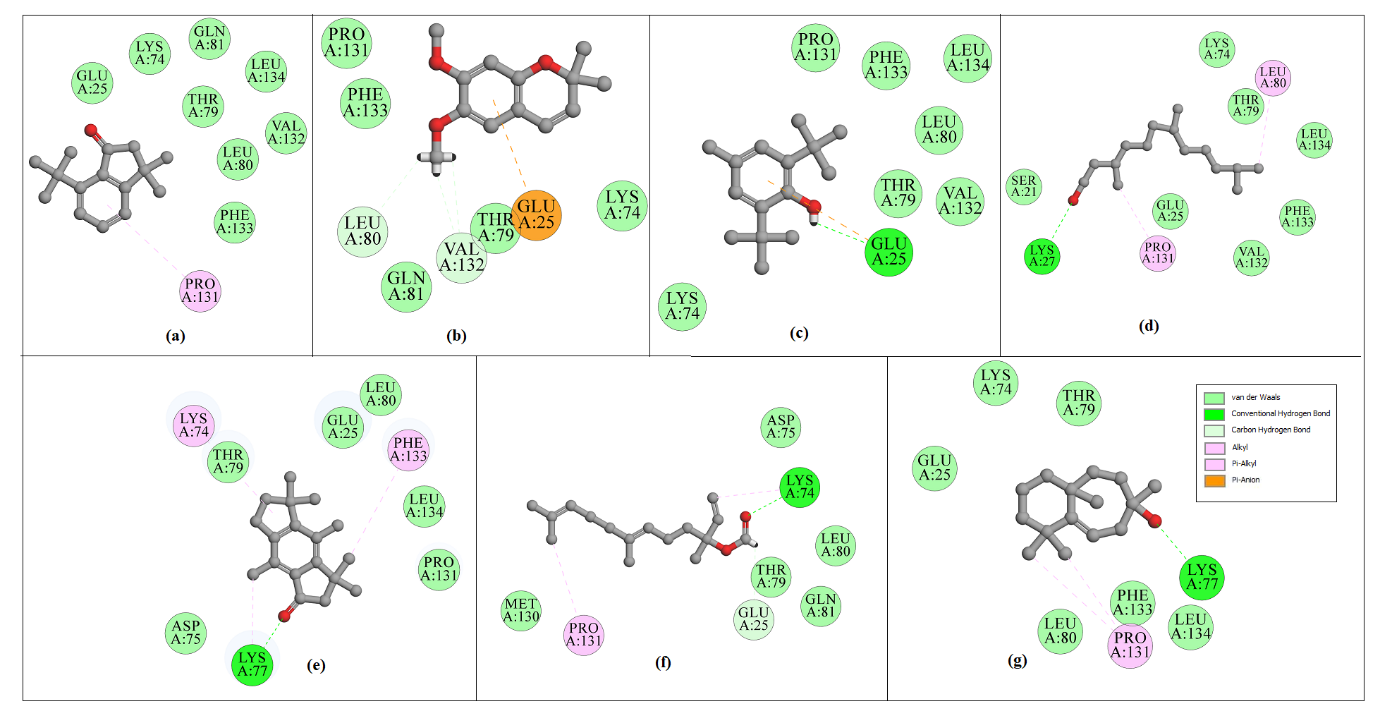


**Fig. SF2** Interaction plot of best hits at the IL1B active site; (a)-(g) representing the 2D interactions of LGA, LGB, LGC, LGD, LGE, LGF and LGG respectively.


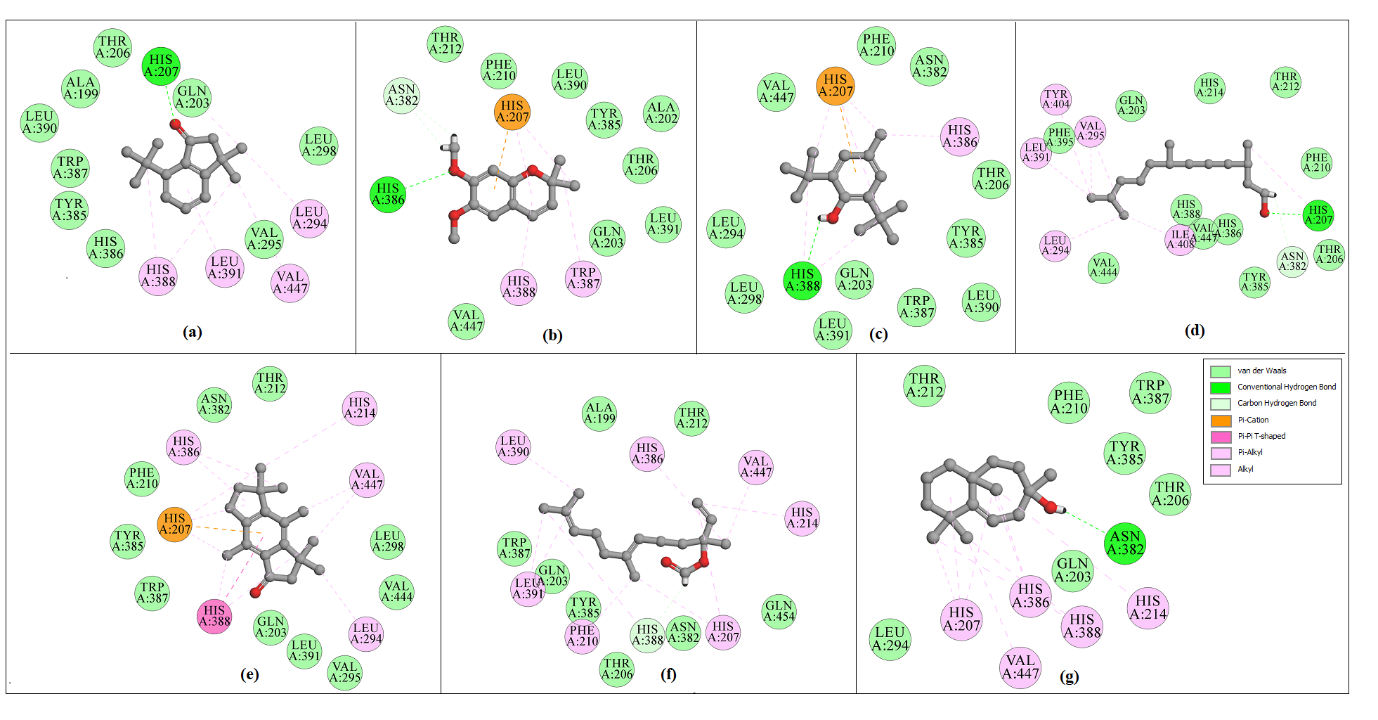


**Fig. SF3** Interaction plot of best hits at the PTGS2 active site; (a)-(g) representing the 2D interactions of LGA, LGB, LGC, LGD, LGE, LGF and LGG respectively.


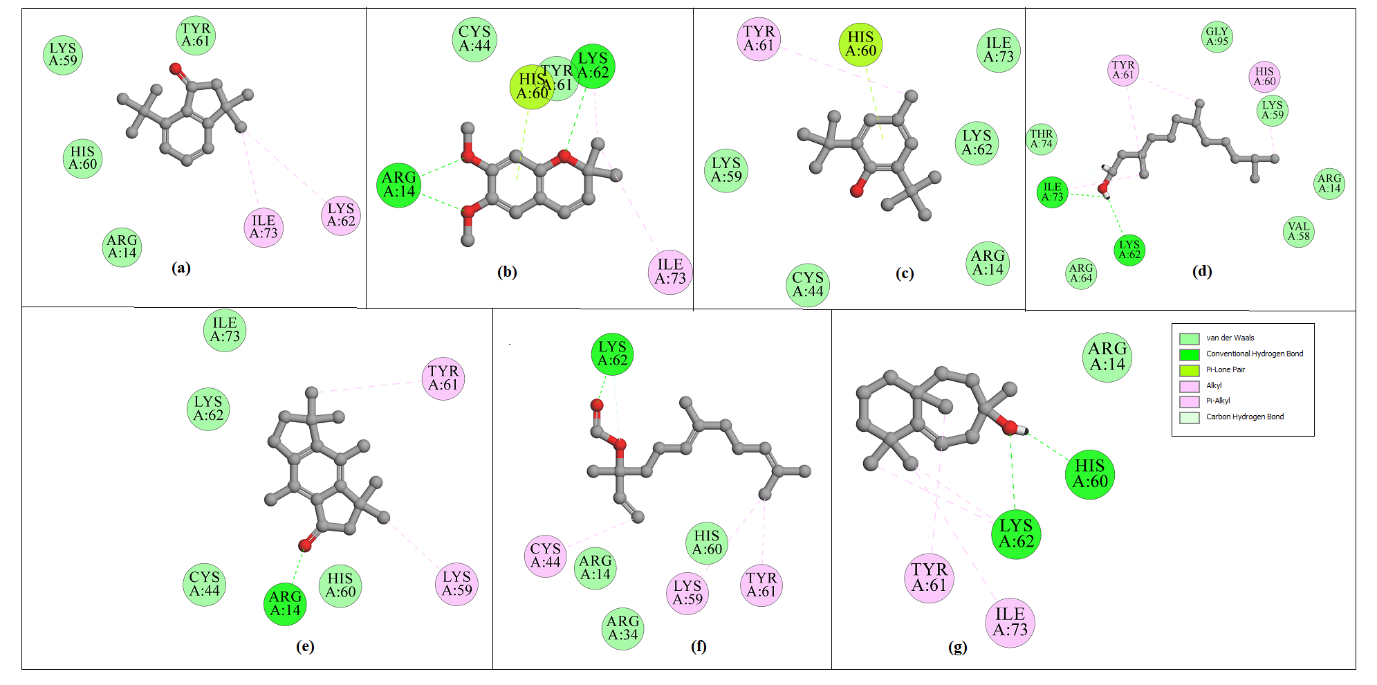


**Fig. SF4** Interaction plot of best hits at the SRC active site; (a)-(g) representing the 2D interactions of LGA, LGB, LGC, LGD, LGE, LGF and LGG respectively.
